# Biomolecular condensates as dynamic regulators of musculoskeletal homeostasis, disease, and therapeutic challenges

**DOI:** 10.1038/s41413-026-00578-6

**Published:** 2026-08-17

**Authors:** Qichen Miao, Ruiqi Gao, Mingyang Huang, Guoqing Wang, Wengang Wang, Ming Luo

**Affiliations:** 1 Department of Spine Surgery and Musculoskeletal Tumor, Zhongnan Hospital of Wuhan University, Wuhan, China; 2 Department of Orthopedics, The First Affiliated Hospital of Zhengzhou University, Zhengzhou, China

**Keywords:** Bone cancer, Bone cancer, Bone

## Abstract

Biomolecular condensates are membraneless assemblies that concentrate proteins, nucleic acids, and other biomolecules into dynamic cellular compartments. Liquid-liquid phase separation (LLPS) is one important route by which such condensates form, particularly when multivalent interactions generate liquid-like, reversible assemblies. However, not every condensate or disease-associated assembly should be explained solely by LLPS. In the musculoskeletal system, condensates have been linked to transcription, signal transduction, RNA metabolism, stress responses, tissue development, homeostasis, and mechanoadaptation. Genetic mutations, altered post-translational modifications, and environmental stress can disturb these assemblies, but the evidence does not always establish whether condensates are causal drivers of disease or downstream responses to injury. This review examines how biomolecular condensates and LLPS-related mechanisms have been implicated in osteoporosis, osteoarthritis, skeletal muscle atrophy, bone and soft tissue sarcomas, and neuromusculoskeletal diseases. We focus on the strength of the available evidence, distinguish correlative observations from causal mechanisms where possible, and discuss how condensates may connect non-coding genetic variants, mechanical cues, metabolic signals, and disease phenotypes. We also assess the translational potential and limitations of condensate-based biomarkers, therapies that target pathological condensates, and phase-separation-inspired delivery systems. A central message is that biomolecular condensates offer a useful framework for musculoskeletal biology, but clinical translation will require disease-specific targets, human-relevant models, selective delivery, and rigorous tests of causality.

## Introduction

The musculoskeletal system includes muscles, bones, ligaments, tendons, cartilage, and other load-bearing tissues. These tissues are connected through specialized interfaces whose mechanical, physical, and biochemical gradients are essential for coordinated movement and tissue homeostasis.^1^ As the body’s principal mechanical framework and mechanosensory network, the musculoskeletal system supports force transmission, locomotion, posture, repair, and metabolic adaptation. Disorders such as osteoporosis, osteoarthritis (OA), and sarcopenia are highly prevalent and constitute a major cause of disability worldwide.^2,3^ Their biology depends on the precise spatial and temporal organization of signaling, transcriptional, metabolic, and stress-response pathways. At the cellular scale, muscle contraction and bone loading are translated into nuclear deformation, cytoskeletal remodeling, and mechano-signaling programs.^4,5^ These processes unfold in a crowded intracellular environment where biomolecules must be organized without depending exclusively on membrane-bound organelles.^6^ Advances in super-resolution imaging, live-cell tracking, and single-molecule analysis have made this organization experimentally accessible and have accelerated the study of liquid-liquid phase separation (LLPS).^7,8^ LLPS provides one route through which biomolecules self-organize into membraneless condensates. It therefore offers a useful framework for linking molecular biophysics to musculoskeletal physiology and disease.

Biomolecular condensates are cellular assemblies that lack surrounding membranes but maintain distinct compositions, dynamics, and functions. LLPS describes a subset of condensate-forming processes in which biomolecules demix into dense and dilute phases, often producing dynamic, liquid-like droplets. This distinction matters because many intracellular bodies exhibit condensate-like behavior without meeting all criteria for equilibrium LLPS, and some pathological assemblies mature into gel-like, solid-like, or amyloid-like states. In cells, condensates contribute to subcellular compartmentalization and cellular function.^9^ For instance, condensates organize promoter and super-enhancer elements during gene regulation.^10,11^ In innate immunity, cyclic GMP-AMP synthase forms DNA-induced condensates that activate antiviral signaling.^12^ In the nervous system, condensate assembly contributes to the postsynaptic density and synaptic signaling.^13,14^ In the cytoskeletal context, phase-separation-related mechanisms localize KANK proteins at focal adhesion edges to modulate actin organization,^15^ and condensates within the mitotic spindle contribute to cell-cycle progression.^16^ Under stress, stress granules (SGs) assemble as condensates that coordinate adaptive cellular responses,^17^ and septins form dynamic liquid-like assemblies involved in cell polarity.^18^ Disease-associated mutations, aberrant post-translational modifications, and age-related stress can alter condensate composition or material state.^19^ Examples include Tau condensation and aggregation in Alzheimer’s disease and disrupted phase behavior of the p53 tetramerization domain in oncogenesis.^20,21^ Despite intense work in cell biology and other disease areas, condensate biology in the musculoskeletal system remains fragmented.^22^ Current evidence links condensate dysregulation to osteoporosis,^23^ osteoarthritis,^24^ bone tumors,^25^ muscle degeneration,^26^ and neurodegenerative diseases that affect neuromuscular function,^27^ but the strength of causal evidence differs across models and diseases.

**Fig. 1:**
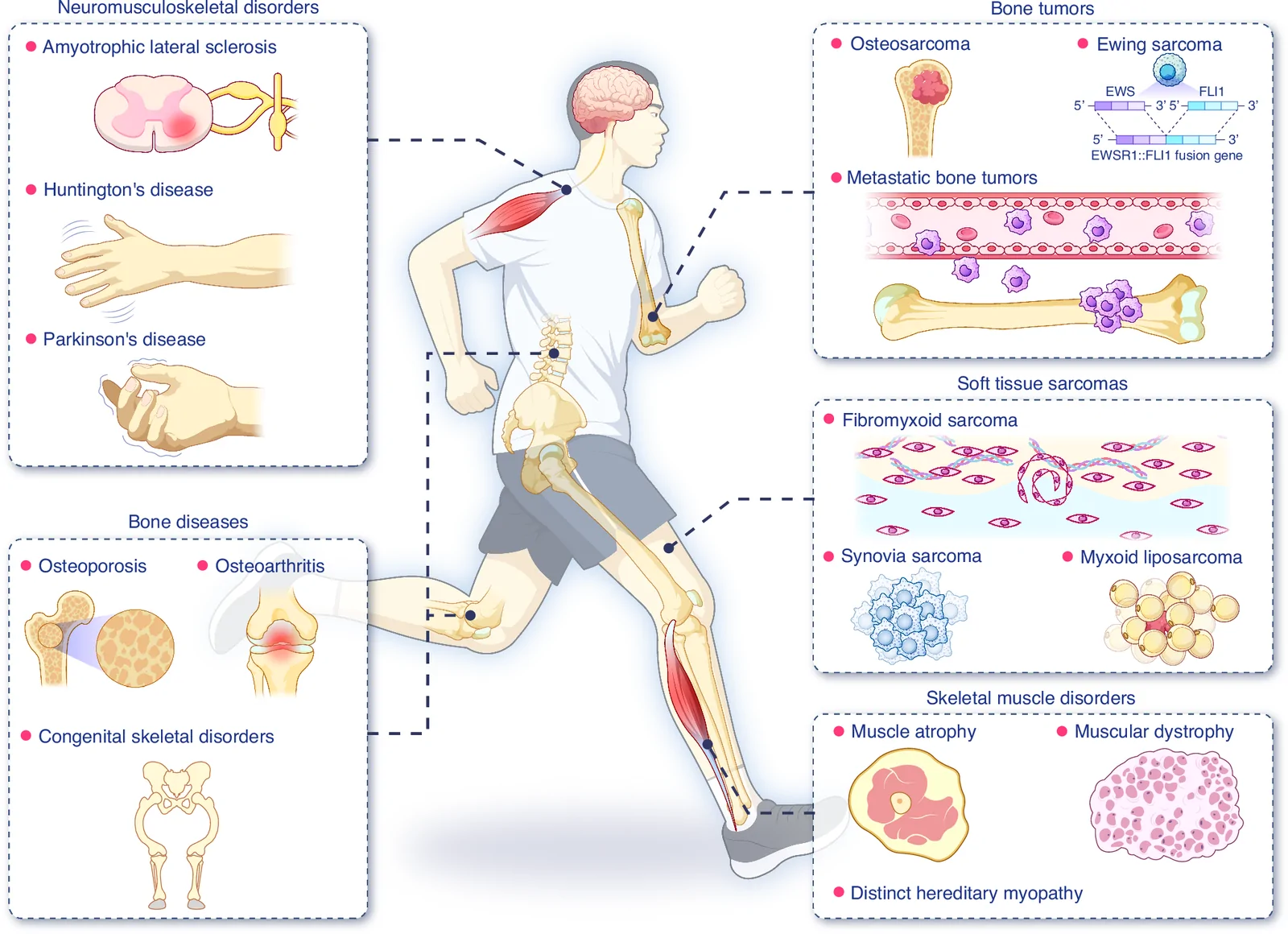
Biomolecular condensates in musculoskeletal diseases. Biomolecular condensates and LLPS-related mechanisms have been implicated in a broad spectrum of musculoskeletal disorders, including neuromuscular diseases, bone pathologies, skeletal conditions, bone tumors, and soft tissue sarcomas

This review synthesizes and critically evaluates current evidence on biomolecular condensates in musculoskeletal diseases, with LLPS discussed as a major but not exclusive mechanism of condensate formation. We address osteoporosis, osteoarthritis, sarcopenia, bone tumors, soft tissue sarcomas, and neuromusculoskeletal disorders (Fig. 1), with attention to the experimental basis for each proposed mechanism. In osteoporosis, RUNX2-related condensates may organize enhancer-promoter contacts and support osteoblast differentiation. In muscular dystrophy, proteins such as hnRNPDL and TDP-43 can shift from dynamic condensates to less reversible assemblies that interfere with RNA processing, autophagy, and myogenic differentiation. In Ewing sarcoma, EWSR1::FLI1 condensates recruit BRG1/BAF complexes to GGAA microsatellites and activate oncogenic transcriptional programs. These examples also illustrate a recurring problem in the field: condensate formation is often observed alongside disease phenotypes, but causality requires perturbations that specifically alter condensate assembly, material state, or client recruitment without globally disrupting protein function. We discuss post-translational modifications, RNA scaffolding, physicochemical conditions, and mechanical inputs that tune condensates in musculoskeletal contexts. We also assess condensate-targeted strategies, including GSK-J4, nilotinib, and biomaterial-based modulation, while emphasizing unresolved issues of target selectivity, tissue delivery, and in vivo validation.

## Biomolecular condensates: formation, material states, and functions

### From liquid-liquid phase separation to biomolecular condensates

Phase behavior has long provided a physical language for describing matter, including the gas, liquid, and solid states of water. In cells, the same physical principles help explain how biomolecules can organize reactions without membrane boundaries. As early as 1899, Edmund Beecher Wilson proposed that cytoplasmic substances might behave as liquids or liquid mixtures with distinct chemical activities.^28^ This idea anticipated a central problem in modern cell biology: how cells create specialized reaction spaces within a crowded cytoplasm and nucleoplasm. Classical organelles, such as the nucleus, endoplasmic reticulum, Golgi apparatus, and lysosomes, solve this problem through lipid membranes and compartment-specific functions.^29^ Membraneless compartments provide a second solution. Structures such as the nucleolus, Cajal bodies, nuclear speckles, and SGs lack lipid bilayers but maintain distinct composition, morphology, and dynamics.^30,31^ Early observations that Cajal bodies behaved like semi-fluid droplets suggested that physical material properties could stabilize such compartments.^32^ A decisive advance came from studies of RNA- and protein-rich P granules in C. elegans embryos, which showed liquid-like behavior and provided experimental and theoretical evidence that LLPS can drive condensate formation.^33^

LLPS is therefore an important mechanism for forming membrane-free cellular structures, but it should be placed within the broader concept of biomolecular condensates. When local concentration and interaction valency exceed a threshold, selected macromolecules can separate from the surrounding dilute phase and form a dense phase with distinct composition and material behavior.^34^ Many condensates show liquid-like exchange, but others become viscoelastic, gel-like, solid-like, or amyloid-like under stress, mutation, or aging. This material-state continuum helps distinguish physiological condensates from irreversible pathological aggregates and explains how normal compartments can harden into toxic assemblies.^35^ Condensate biology therefore integrates thermodynamics, kinetics, molecular interactions, and cellular regulation across transcription, RNA metabolism, signaling, and stress adaptation.^36^ Known membraneless organelles implicated in musculoskeletal diseases are summarized in Table 1.

**Table 1:** Known membraneless organelles in musculoskeletal system diseases

Membraneless organelle	Key molecules	Mechanism	Function	Reference
Paraspeckles	MATR3 and PABPN1	The PABPN1-MATR3 functional axis regulates paraspeckles through Neat1	Maintain the normal number of paraspeckles and mediate the nuclear retention and editing of specific transcripts	^171^
	MEN ε/β and lncRNA	MEN ε/β lncRNA functions as a “scaffold” to assemble paraspeckles	Regulate muscle cell differentiation	^166^
	Neat1	Mechanical stimulation upregulates the expression of lncRNA Neat1, promoting the assembly of paraspeckles	Inhibit Smurf1 mRNA translation via sequestration to enhance osteogenic differentiation	^97^
Nuclear speckles	DGK-ζ	DGK-ζ interacts with PLCβ1 in the nucleolus to regulate nuclear lipid signaling	Regulate the process of skeletal muscle cell differentiation	^167^
	PABPN1	Mutant PABPN1 aberrantly accumulates at the periphery of nuclear speckles to form inclusions	Cause post-transcriptional defects that propel myofiber pathology	^170^
Stress granules	HuR	The RNA-binding protein HuR undergoes LLPS to incorporate into stress granule condensates	Protect β-catenin mRNA to promote osteogenic differentiation	^23^
	TIA1	IL-1β stimulation induces the assembly of stress granules in osteoarthritic chondrocytes	Delay COX-2 mRNA translation to control inflammation and cartilage degradation	^24^
	TDP-43 and p35	TDP-43 is cleaved to generate a 35 kD fragment that promotes stress granule formation	Anti-inflammatory, promotes proliferation, and enhances the anti-apoptotic ability of chondrocytes	^111^
	GRP78	The chaperone protein GRP78 induces stress granule formation	Promote drug-resistant survival and bone metastasis recurrence	^133^
	SQSTM1 and TIA1	The TIA1-N357S variant enhances LLPS and inhibits the autophagic clearance of stress granule dynamics	Impaired stress granule clearance contributes to myotoxicity.	^174^
P-bodies	YTHDF2	m^6^A-modified TM4SF1 mRNA induces LLPS of YTHDF1-3, promoting the formation of P-bodies	Degrade TM4SF1 mRNA to regulate skeletal muscle development	^90^
	SYK	SYK localizes to P-bodies during TGF-β-induced epithelial-mesenchymal transition	Restore epithelial phenotype and promote tumor bone metastasis colonization	^135^
Nucleus	HMGB1	Frameshift mutations in HMGB1 cause abnormal LLPS and lead to nucleolar abnormalities	Cause multiple system developmental malformations	^113^

*LLPS* liquid-liquid phase separation

### Formation mechanisms and experimental evidence for condensates

Multivalent weak interactions are a principal driver of LLPS. Individually, these contacts between proteins, nucleic acids, or both are weak and transient, but collectively they generate cooperative networks that can stabilize condensates.^19,37^ Once concentration, valency, or interaction strength crosses a threshold, a dense phase can form alongside a dilute phase.^38–41^ This threshold behavior explains why small changes in protein abundance, RNA binding, post-translational modification, or environmental conditions can markedly alter condensate assembly.^42^ Evidence for LLPS, however, requires careful interpretation. 1,6-hexanediol can perturb condensates, but it can also affect membranes, chromatin, and hydrophobic interactions; its use alone does not prove LLPS.^43^ Similarly, purified protein assays are valuable for defining phase behavior, but they may not reproduce the crowded, mechanically loaded, and matrix-rich environments of bone, cartilage, or skeletal muscle. Stronger evidence generally requires convergent tests, including endogenous tagging, concentration-dependent phase diagrams, FRAP or single-particle tracking, valency-disrupting mutations, rescue experiments, and disease-relevant in vivo or human-cell models.

Intrinsically disordered regions (IDRs) are major molecular elements that enable multivalency. These regions have low sequence complexity and high conformational flexibility, are enriched in selected amino acids, and lack stable tertiary structure.^44,45^ Their flexibility allows multiple weak interaction sites to engage simultaneously, while repetitive or low-complexity motifs increase valency.^44,46^ IDRs are also sensitive to pH, ionic strength, post-translational modifications, and pathogenic mutations. For example, D378N/H mutations in the prion-like domain of hnRNPDL disrupt electrostatic balance, accelerate aggregation, impair function, and cause limb-girdle muscular dystrophy type 1 G.^46–48^ Short-range interactions within IDRs can form transient local structures that further shape phase behavior, as shown by the C-terminal alpha-helix that supports TDP-43 LLPS.^49,50^ Folded domains complement IDRs by providing stable binding surfaces, nucleation points, and higher-order scaffolding.^51–53^ Nucleophosmin illustrates this synergy: its N-terminal folded domain mediates pentamerization, which enables subsequent IDR-driven phase separation during client binding.^54,55^

Other sequence features also tune condensate assembly. Low-complexity domains (LCDs), which are enriched for a limited set of amino acids, often overlap functionally with IDRs and are strongly influenced by the intracellular environment. The saturation concentration for phase separation can depend on the combined contributions of tyrosine residues in prion-like domains (PrLDs) and arginine residues in arginine-rich RNA-binding regions.^56^ In addition to tyrosine-arginine interactions, arginine can promote phase separation through arginine-arginine contacts and heterotypic interactions with residues such as glutamine and serine. Polar residues add further specificity: glutamine can stabilize condensates through sequence-specific contacts, whereas glycine increases backbone flexibility and facilitates favorable neighboring interactions.^57,58^ These findings extend the classical stickers-and-spacers model by showing that condensate formation arises from a distributed interaction network rather than from a small set of sticker residues alone.

### Regulation of condensate assembly and material properties

**Fig. 2:**
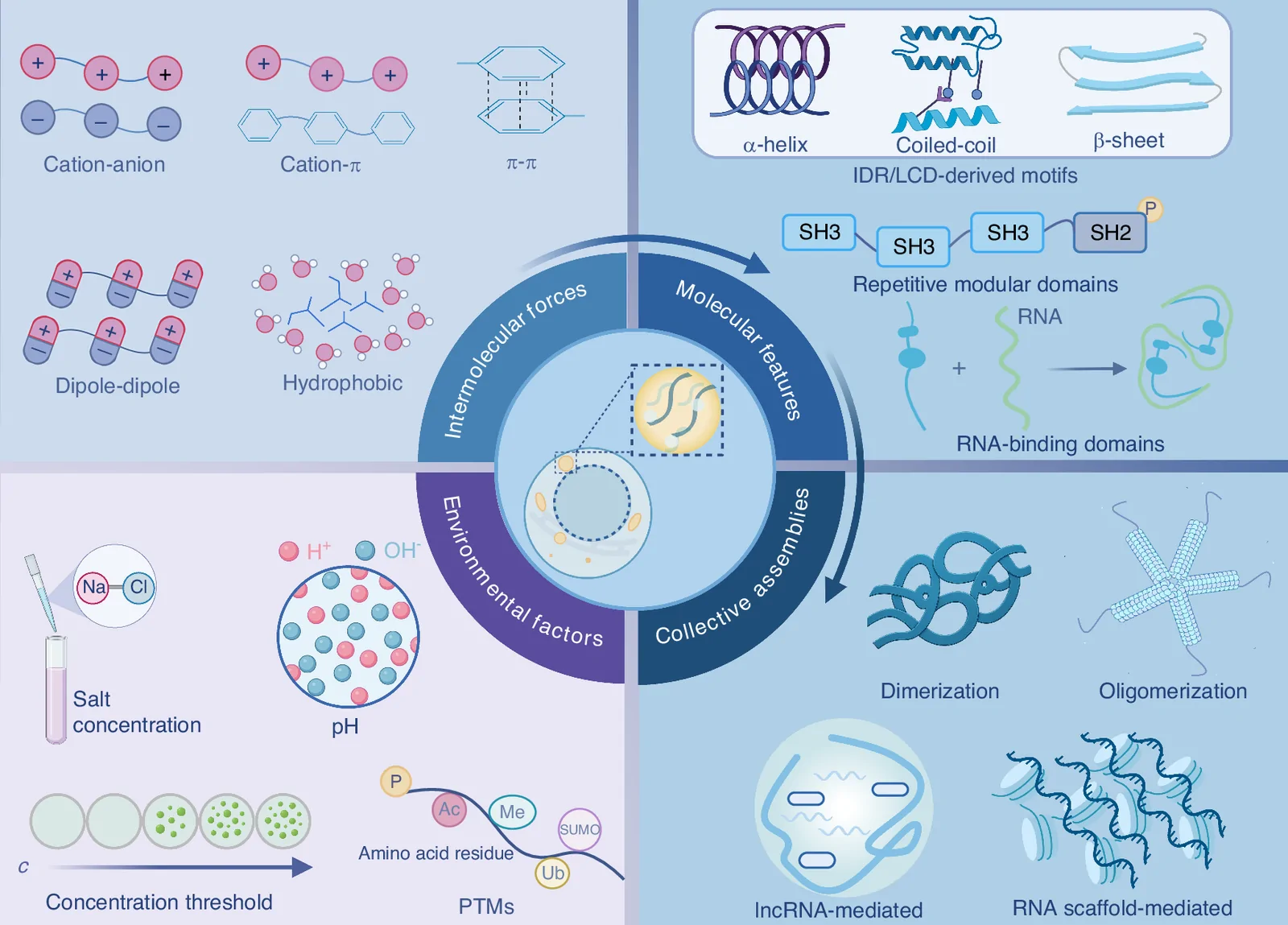
Assembly mechanisms of biomolecular condensates. Biomolecular condensates can form through networks of weak, multivalent intermolecular interactions, including cation-anion interactions, cation–π interactions, π–π stacking, dipole-dipole interactions, and hydrophobic forces. These interactions are underpinned by characteristic molecular features that confer multivalency, including intrinsically disordered regions/low-complexity domains, repetitive modular domains, and RNA-binding domains. Such features promote higher-order collective assemblies and can support LLPS when dense and dilute phases form. Environmental cues, such as salt concentration, pH, protein or RNA concentration thresholds, and post-translational modifications, dynamically regulate condensate initiation, stability, and material properties

Condensate assembly is dynamically regulated by nucleic acids, post-translational modifications, metabolites, and physicochemical parameters. These factors tune the assembly, dissolution, material properties, and biological output of phase-separated compartments (Fig. 2).

#### RNA: from core scaffolds to bidirectional regulators

The components of LLPS systems are often described as scaffolds and clients.^59^ RNA can act as a scaffold by recruiting and concentrating RNA-binding proteins. For example, m^6^A-modified RNA promotes YTHDF2 recruitment and phase separation.^60^ RNA can also define condensate identity and prevent inappropriate droplet fusion. Whi3 forms distinct condensates with different mRNAs; droplets carrying different mRNAs do not readily fuse, whereas droplets carrying the same mRNA can coalesce.^61^ RNA sequence and length can further influence material transitions. Homopolymeric RNA aggregation can promote liquid-to-solid transitions in multicomponent condensates, a process linked to Huntington’s disease and ALS.^62^

RNA regulates LLPS in a concentration-dependent and context-dependent manner. At low concentrations, mRNA can support transcriptional condensates through electrostatic interactions with positively charged MED1.^63^ At excessive concentrations, RNA can instead inhibit FUS phase separation.^63,64^ Beyond these general effects, lncRNAs provide more specialized control of chromatin-associated condensates and gene expression.^65^ In skeletal muscle, lncRNAs influence proliferation and differentiation by modulating condensate behavior. For example, lnc-RMG regulates myogenic phase separation by producing miR-133a-3p, reducing MEIS2 abundance, and relieving transcriptional repression of TGF-β signaling.^66^ Similarly, maternally expressed gene 3 supports SUZ12 phase separation and stabilizes Polycomb repressive complex 2, thereby limiting atrophy-associated and lipid-accumulation programs and preserving muscle quality and metabolic balance.^67^

#### Post-translational modifications: chemical switches for fine-tuning phase separation

Post-translational modifications add a major regulatory layer by changing protein charge, conformation, interaction interfaces, and valency.^68^ Phosphorylation is especially common in IDRs and introduces negatively charged phosphate groups that can weaken or reorganize electrostatic interactions.^69^ For example, phosphorylation of p53 at S392 reduces intermolecular interactions within droplets and increases droplet fluidity.^70^ Acetylation can also regulate phase behavior; acetylation of the autophagy-related protein RB1CC1 modulates its phase separation and influences autophagosome formation.^71^ Arginine methylation alters cation-pi interaction capacity and can reduce hnRNPA2 phase separation by disrupting arginine-mediated contacts.^72^ SUMOylation, ubiquitination, ADP-ribosylation, and other modifications introduce additional multivalent interaction modules and thereby reshape condensate assembly and function.^68,69,73^

#### Sensitive modulation by physicochemical parameters

**Fig. 3:**
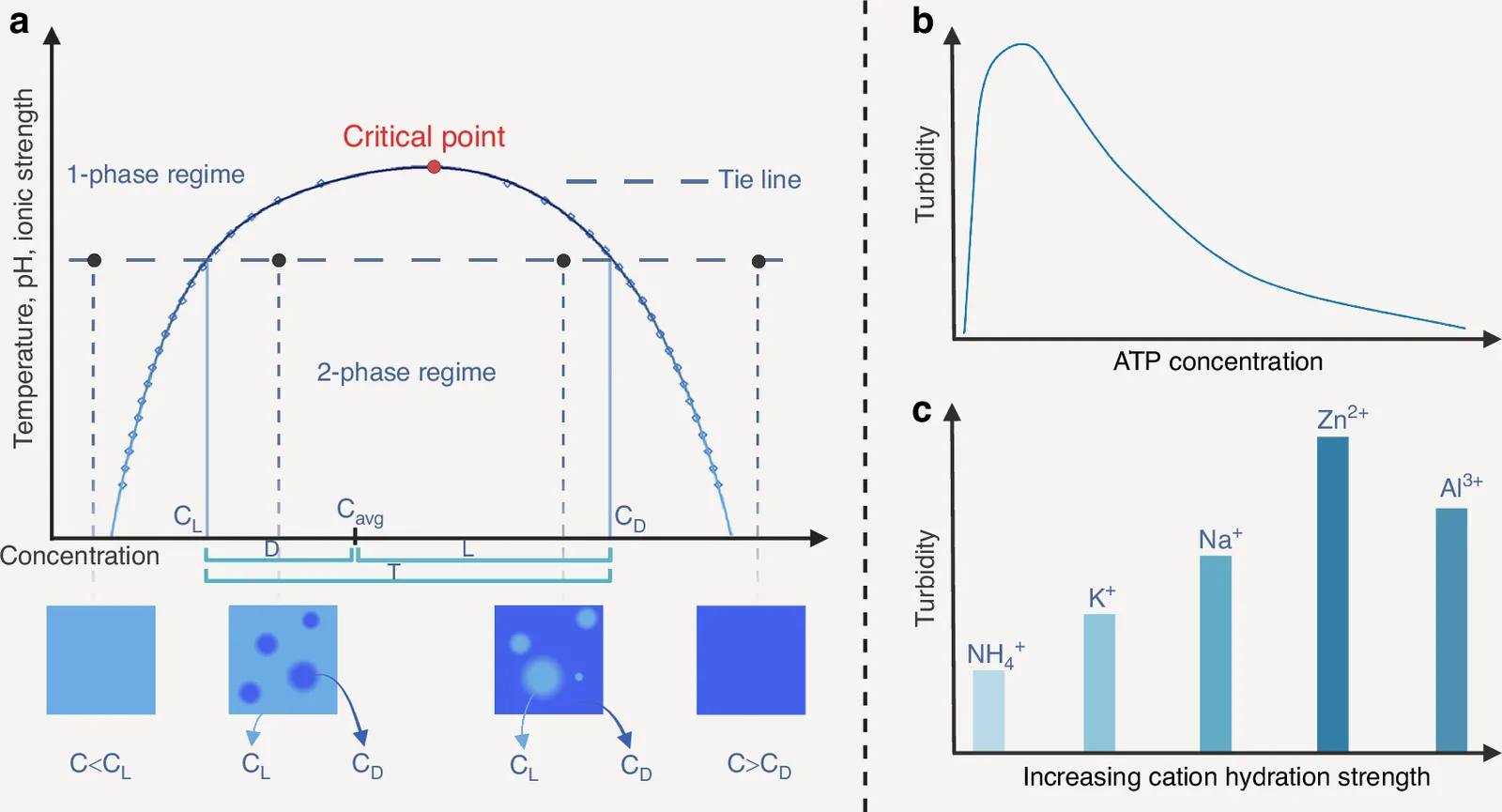
Phase diagram and regulatory principles of liquid-liquid phase separation. **a** The binodal (coexistence) line in the phase diagram, governed by factors such as temperature and pH, separates the one-phase and two-phase regimes. Within the two-phase regime, LLPS produces a dilute phase with concentration *C*_L_ and a dense phase with concentration C_D_. At fixed temperature and pH, *C*_L_ and *C*_D_ remain constant, whereas the volume fractions of the dilute (*f*_L_) and dense (f_D_) phases vary with the system’s average concentration (*C*_avg_), following the lever rule: *f*_L_ = (*C*_D_ − *C*_avg_)/(*C*_D_ − *C*_L_) and *f*_D_ = (*C*_avg_ − *C*_L_)/(*C*_D_ − C_L_). **b** Representative example showing the biphasic effect of ATP on the turbidity of the prion-like domain of TDP-43. Low ATP concentrations promote liquid-liquid phase separation and increase turbidity, whereas high concentrations dissolve phase-separated droplets and suppress phase separation. **c** Schematic illustrating the effects of different cations on Tau condensate formation. Cations are ordered by increasing hydration strength, reflecting their varying capacities to modulate protein phase separation

The intracellular microenvironment is tightly regulated, and small changes in physicochemical parameters can strongly influence LLPS dynamics (Fig. 3). Increased osmotic pressure compresses cell volume and alters hydration, molecular concentration, and molecular crowding, thereby favoring phase separation. Temperature also regulates LLPS; even changes of about 1 °C can trigger droplet coalescence or dissolution in proteins such as BuGZ, DEAD-box helicase 4, hnRNPA1, and FUS, illustrating the sensitivity of condensate assembly.^22^ pH affects electrostatic attraction and repulsion by altering protonation states of charged residues in proteins or RNA. For example, the IDR of galectin-3 regulates self-assembly through pH-dependent protonation, promoting aggregation at neutral pH and ligand dissociation at acidic pH.^74^ Ionic strength primarily acts through charge screening. Low ionic strength favors electrostatic attraction, whereas higher ionic strength often weakens these interactions and suppresses LLPS.^75^ Cation identity also matters. Highly hydrated cations such as Zn^2+^ and Al^3+^ can promote Tau phase separation even at high ionic strength, probably by altering solvent-water structure and dynamics rather than by direct coordination alone.^76^

#### ATP: a concentration-dependent bifunctional regulator

ATP, beyond its role as an energy currency, functions as a bidirectional regulator of LLPS.^77,78^ Using TDP-43 as an example, ATP at low concentrations promotes LLPS by engaging arginine residues in the PrLD of TDP-43 via its hydrophobic purine ring and negatively charged triphosphate chain. This bivalent interaction synergistically enhances hydrophobic effects, strengthening intermolecular attraction and supporting droplet formation. Conversely, at high concentrations, ATP saturates arginine binding sites, coating the PrLD with negatively charged triphosphate groups. This introduces long-range electrostatic repulsion and disrupts hydrophobic interactions essential for condensate integrity, resulting in dissolution.^79^ The same phenomenon can also occur with FUS protein.^80,81^

#### External signals: connecting cellular stress to adaptive responses

In addition to endogenous metabolites and intracellular microenvironmental regulation, condensate assembly helps cells respond rapidly to external signals. Oxidative stress, heat shock, and extracellular mechanical stiffness can alter condensate formation and material properties. For example, arsenic-induced oxidative stress drives YTHDF2 phase separation, a process that contributes to arsenic toxicity.^82^ Heat shock can induce LLPS of NEDD4-binding partner 1, which protects cells under stress conditions.^83^ The mechanical and physical environment also regulates condensates. In Drosophila muscle, Tono acts as a mechanically sensitive transcriptional regulator. It detects compressive forces generated by myofibril and mitochondrial growth within the muscle nucleus, triggering LLPS to form nucleoplasmic droplets that regulate developmental transcriptional switches, sarcomere maturation, and mitochondrial functionality.^84^

Condensate assembly is a cooperative process driven by multivalent weak interactions and regulated by IDRs, low-complexity domains, folded domains, RNAs and their modifications, post-translational modifications, metabolites, and the cellular microenvironment. LLPS is a useful mechanistic model when these interactions produce liquid-like demixing, but condensates can also acquire more complex material states. This framework couples molecular structure with cellular function and creates vulnerabilities that may contribute to disease. Clarifying the molecular rules that define condensate formation, maturation, and dissolution may support therapeutic strategies that modulate pathological condensates without disrupting physiological ones.

### Core functions of biomolecular condensates in the musculoskeletal system

**Fig. 4:**
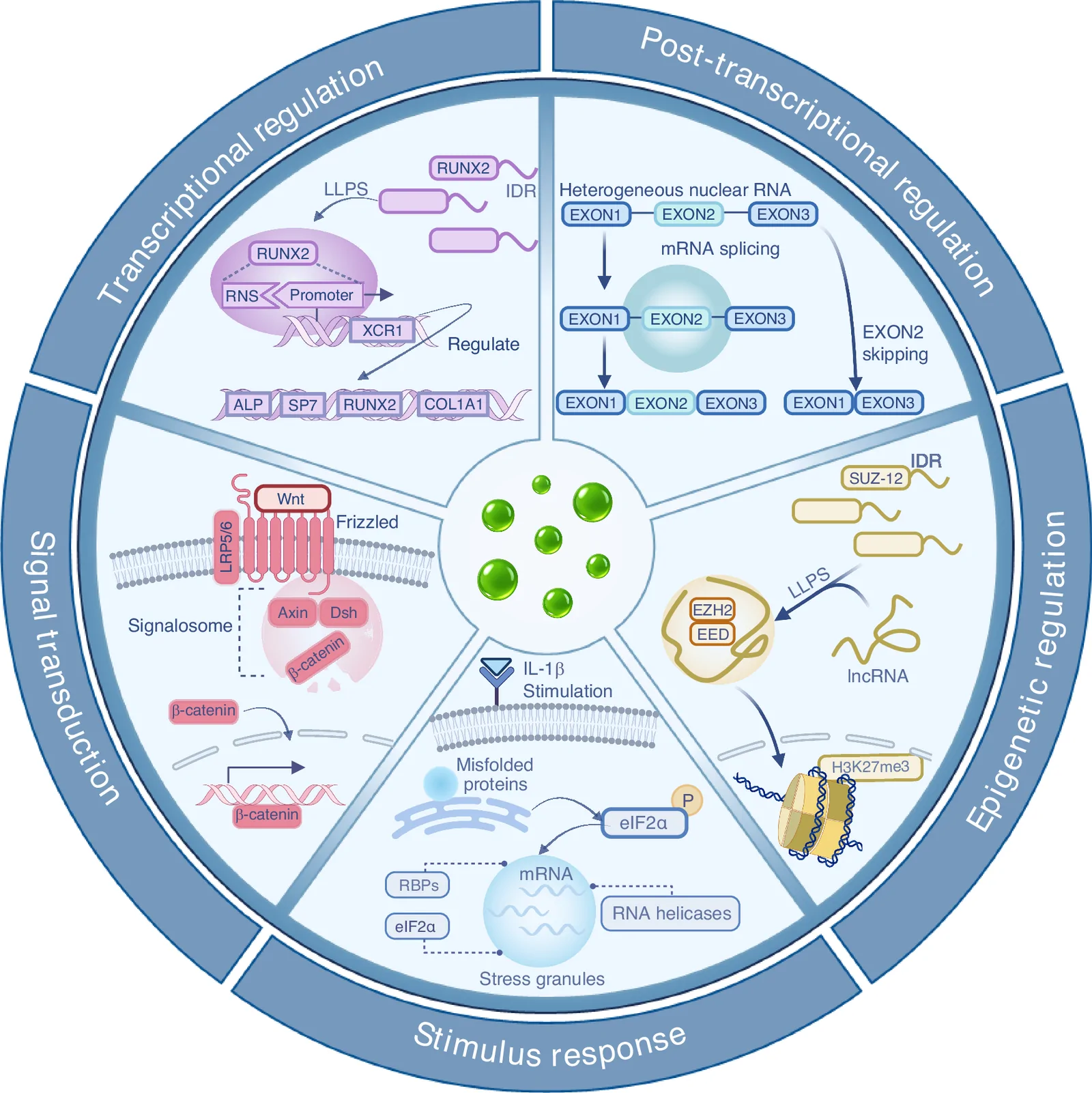
Representative regulatory roles of biomolecular condensates in musculoskeletal biology. Transcriptional regulation: RUNX2 forms condensates that bridge the rs4683184-containing enhancer and promoter regions, supporting the assembly of transcriptional machinery and activation of downstream genes. Post-transcriptional regulation: Condensates contribute to alternative splicing and exon inclusion; their specific roles in musculoskeletal tissues remain to be fully defined. Signal transduction: Wnt activation promotes the degradation of phase-separated signalosome condensates, releasing β-catenin for nuclear translocation and target gene regulation. Epigenetic regulation: SUZ12- and lncRNA-associated condensates selectively recruit EZH2 and EED, promoting Polycomb-mediated histone modifications within the nucleus. Cellular stress response: Accumulation of misfolded proteins at the endoplasmic reticulum triggers eIF2α-mediated signaling and promotes SG assembly

After describing condensate formation and regulation, we now focus on the cellular roles and physiological functions of condensates in the musculoskeletal system. Biomolecular condensates contribute to transcriptional regulation, post-transcriptional regulation, signal transduction, epigenetic regulation, and stimulus responses (Fig. 4). In some examples, the underlying mechanism has been directly linked to LLPS; in others, the safer interpretation is that condensate organization is involved, with the precise material mechanism still requiring validation.

#### Transcriptional regulation

Within the musculoskeletal system, one well-studied role of biomolecular condensates is transcriptional regulation. Condensates can either activate or repress transcription depending on their composition and recruited clients. During muscle proliferation, TAZ forms dynamic nuclear condensates through phase-separation-related mechanisms and recruits Smad7 and β-catenin, thereby inhibiting myogenic gene expression.^85^ Conversely, transcriptional condensates can concentrate core transcription factors, coactivators, and RNA polymerase II into local hubs that activate gene programs.^86,87^ In osteoblasts, RUNX2 phase separation bridges the rs4683184 enhancer and the XCR1 promoter, promoting osteogenic differentiation.^88^ In skeletal muscle, MEF2D undergoes beta-domain-mediated LLPS and liquid-to-solid transition to form spatially segregated transcriptional hubs that recruit basal transcription machinery and myogenic factors during staged muscle development.^89^

#### Post-transcriptional regulation

Biomolecular condensates also regulate post-transcriptional gene expression by controlling mRNA localization, stability, and translation. They can sequester selected mRNAs and inhibit translation. For example, YTHDF2 undergoes LLPS and transports N6-methyladenosine (m^6^A)-modified TM4SF1 mRNA to P-bodies for degradation, thereby reducing TM4SF1-mediated inhibition of myocyte differentiation.^90^ In myopathy, abnormally accumulated cellular prion protein (PrPᶜ) undergoes phase separation after binding specific miRNAs. These miRNA-bound condensates target the 3’ UTRs of ATG genes and inhibit autophagy-related translation.^26^ Condensates can also protect transcripts from degradation. Nucleolin-based condensates stabilize HIF-1α mRNA and increase protein expression.^91^ Current musculoskeletal studies mainly address mRNA localization, protection, and degradation, whereas condensate roles in RNA modification, splicing, editing, and translation remain less defined.^26^

#### Signal transduction

Biomolecular condensates participate in signal transduction by concentrating pathway components in space and time. During TGF-beta signaling, disheveled-binding antagonist of β-catenin 1 forms cytoplasmic LLPS-driven condensates that enrich CK2 and inhibit Wnt/β-catenin signaling.^92^ At the neuromuscular junction, Rapsyn drives LLPS through multivalent tetratricopeptide-repeat interactions, forming dynamic condensates that recruit acetylcholine receptors, cytoskeletal proteins, and signaling molecules to support synaptic transmission.^93^ During early muscle injury, 11,12-epoxyeicosatrienoic acid diffuses into the repair microenvironment and amplifies FGF-FGFR signaling by enhancing fibroblast growth factor condensation.^94^

#### Epigenetic regulation

In musculoskeletal tissues, LLPS-linked condensates also contribute to epigenetic regulation. During muscle development, maternally expressed gene 3 promotes SUZ12 LLPS and stabilizes Polycomb repressive complex 2. This enhances H3K27me3 at the Fhl3 and Rnf128 loci, limits fast-twitch fiber transition and fat infiltration, and helps preserve oxidative muscle function and mass.^67^ Condensates can also repress transcription through physical compartmentalization. In myoblasts, the C-terminal IDR of MEIS2 mediates LLPS and forms nuclear condensates that bind the transforming growth factor-beta receptor II promoter and inhibit transcription.^66^ Similarly, lethal (3) malignant brain tumor-like protein 2 forms nuclear condensates through its C-terminal IDR and polybasic region. These condensates recruit repressive components and sequester the promoter of interferon-induced protein with tetratricopeptide repeats 2, suppressing oncogenic transcription and exerting tumor-suppressive activity.^95^

#### Stimulus response

Cells use condensate assembly to respond quickly to external stimuli. SGs are a classic example because they assemble rapidly during stress and support cytoprotection.^96^ In human OA chondrocytes, IL-1β induces endoplasmic reticulum stress and eIF2α phosphorylation, leading to SG assembly. These SGs sequester COX-2 mRNA and delay inflammatory protein production, thereby buffering premature inflammatory responses.^24^ Paraspeckles are also mechanosensitive. In osteoblasts, the lncRNA NEAT1 responds to mechanical stimulation and supports morphological and functional adaptation of paraspeckles under altered gravitational conditions.^97^ In muscle, hypoxia triggers nucleolin translocation from the nucleus to the cytoplasm and reduces its phase-separation capacity, thereby altering gene-expression control.^91^

Together, biomolecular condensates provide spatial and temporal control over musculoskeletal development, homeostasis, and stress adaptation. They coordinate epigenetic regulation, transcription, RNA metabolism, signal transduction, and stimulus responses. Important gaps remain, including how condensate size, composition, fluidity, and material state determine function in specific musculoskeletal cell types. Future work should also move beyond isolated condensates and examine how multiple condensates compete for clients, exchange signals, and respond collectively to mechanical cues. Because current musculoskeletal studies focus heavily on transcriptional regulation, additional work is needed to define condensate functions at post-transcriptional, metabolic, tissue, and organismal levels.

## Biomolecular condensates in musculoskeletal disorders

Biomolecular condensates organize cellular biochemistry, and their dysregulation has been associated with several bone and musculoskeletal diseases. The strength of this association varies across disease settings. In some sarcomas, fusion proteins or transcription factors form condensates that recruit chromatin regulators and activate oncogenic programs, providing relatively direct mechanistic links. In osteoarthritis, SGs correlate with inflammatory signaling and chondrocyte survival, but whether they initiate disease or buffer injury-induced stress remains less certain. In osteoporosis and muscle atrophy, condensate-related mechanisms connect genetic variation, RNA metabolism, mechanical loading, and cell differentiation, yet many observations still require causal validation in human-relevant systems. Here, we review biomolecular condensates in bone diseases, bone tumors, soft tissue sarcomas, skeletal muscle disorders, and neuromuscular disorders, focusing on both mechanistic insights and the limits of current evidence.

### Biomolecular condensates in bone disease

Bone provides mechanical protection, supports muscle attachment, and converts muscle contraction into movement through load-bearing architecture.^98^ Bone diseases disrupt these functions through degenerative, inflammatory, developmental, or metabolic mechanisms and can cause disability, reduced quality of life, complications, and substantial health-care burden.^99,100^ Condensate biology is relevant because osteoblast differentiation, chondrocyte stress responses, mechanical loading, and RNA regulation all depend on spatially organized molecular assemblies. The following sections summarize condensate-related mechanisms in osteoporosis, osteoarthritis, and congenital skeletal disorders (Table 2).

**Table 2:** Biomolecular condensates in bone diseases

Disease	Subcellular localization	Core molecule	Recruited factors	Phenotype	Function
Osteoporosis^23^	Cytoplasm	HuR	β-catenin, G3BP1, TIA1	Decreased bone mineral density, reduced bone formation, and impaired osteogenic differentiation	Enrich and stabilize β-catenin mRNA, recruit β-catenin
Osteoporosis^88^	Nucleus	RUNX2	Enhancer element, XCR1 promoter	Impaired osteogenic differentiation and decreased mineralization capacity	Mediate long-range chromatin interactions between enhancers and promoters, activate XCR1 gene transcription
Osteoporosis^104^	Cytoplasm	Plectin	Annexin A2	Promote osteoblast differentiation and reverse bone loss	Enrichment and activation of osteogenic protein Annexin A2
Osteoarthritis^24^	Cytoplasm	TIA1, HuR	COX-2 mRNA	COX-2 protein expression is delayed but persistently enhanced, PGE2 secretion is increased	Sequester COX-2 mRNA and delay its translation
Osteoarthritis^111^	Cytoplasm	TDP-43	RACK1	Increased chondrocyte proliferation, decreased secretion of inflammatory factors, inhibited apoptosis	Prevent RACK1 from activating the downstream MTK1–MKK3/6–p38/JNK signaling pathway
BPTAS^113^	Nucleus	HMGB1	FIB1	Brachydactyly, polydactyly, tibial hypoplasia, craniofacial anomalies	Induce nucleolar stress, inhibit rRNA transcription and protein translation

*BPTAS* brachyphalangy, polydactyly and tibial aplasia/hypoplasia syndrome

#### Osteoporosis

Osteoporosis is a chronic metabolic bone disease characterized by reduced bone mineral density, deterioration of bone microarchitecture, and increased fracture susceptibility. It causes pain, limits activity, and increases disability risk. A central challenge is to identify mechanistic biomarkers that can be translated into clinical practice.^101^ Biomolecular condensates formed through LLPS may provide such biomarkers because they report functional molecular states rather than protein abundance alone.^102^

At the genetic level, osteoporosis risk loci identified by genome-wide association studies are often located in non-coding regions whose functions are difficult to interpret.^103^ RUNX2 condensates provide one mechanistic example. Through LLPS, RUNX2 can bridge the rs4683184 enhancer and the distal XCR1 promoter, activate GPCR signaling, and promote osteoblast differentiation.^88^ Condensates also regulate mRNA fate in osteoblasts. HuR forms SGs that capture and stabilize beta-catenin mRNA during aging, thereby preserving osteogenic differentiation; loss of HuR granules contributes to functional decline.^23^ NEAT1-dependent paraspeckles retain Smurf1 mRNA in the nucleus under mechanical loading, stabilizing RUNX2 and supporting osteocyte differentiation. Unloading or aging decreases NEAT1 expression, disrupts paraspeckles, and contributes to osteoporosis.^97^ A cytoskeletal mechanism has also been described: plectin undergoes IDR-mediated LLPS and sequesters annexin A2, enhancing osteogenic gene expression and bone formation in vitro and in vivo. The phase-separating plectin region (residues 1967-2185) alleviated bone loss in ovariectomized and mechanically unloaded mice.^104^ These findings suggest that condensates integrate genetic, RNA, cytoskeletal, and mechanical inputs during bone formation.

The osteoporosis literature shows that condensates can mediate distinct and sometimes opposite RNA outcomes, including nuclear retention, translational repression, transcript stabilization, and cytoplasmic signaling. The rules that determine these outcomes remain unclear. NEAT1 illustrates this complexity. In osteoblasts, altered mechanical loading can also increase NEAT1 expression, allowing NEAT1 to sponge miR-466f-3p, derepress HK2, activate autophagy, and potentially promote osteoporosis.^105^ In osteoclasts, differentiation stimuli upregulate NEAT1 in a dose-dependent manner, and excessive activation can reduce bone mass and increase osteoporosis risk.^106^ Future work should determine when NEAT1-containing condensates protect bone and when they promote bone loss. The mechanisms connecting mechanical force, NEAT1 expression, paraspeckle integrity, and LLPS also require clarification. BMP2-inducible kinase, which is associated with bone differentiation and forms cytoplasmic condensates, is another example whose downstream pathway remains insufficiently defined.^107^

#### Osteoarthritis

OA causes progressive joint pain, stiffness, restricted mobility, deformity, and secondary muscle atrophy, substantially reducing quality of life.^108^ Existing treatments do not reliably halt disease progression, so mechanisms that control chondrocyte stress responses remain important therapeutic targets.^109^ Biomolecular condensates, especially SGs, have therefore become a relevant focus in OA research. Transcriptomic analyses using machine learning have also identified condensate-related genes as potential diagnostic biomarkers linked to immune imbalance and chondrocyte degeneration.^110^

In OA, SGs appear to regulate inflammatory responses and chondrocyte survival through a time-dependent sequestration-and-release mechanism. In IL-1β-stimulated human OA chondrocytes, IL-1β induces eIF2α phosphorylation and SG assembly. HuR binds COX-2 mRNA and recruits it into SGs, where the transcript is stabilized but temporarily withheld from translation. COX-2 protein synthesis increases only after SG disassembly and mRNA release, making SGs post-transcriptional regulators of the IL-1β-COX-2 pathway.^24^ SGs also regulate apoptotic and stress signaling. A p35 fragment of TDP-43 promotes RACK1 expression and recruitment into SGs, limiting activation of the pro-apoptotic and pro-inflammatory p38/JNK MAPK pathway. This attenuates inflammation and apoptosis in chondrocytes and may support proliferation through pathways such as LIMK1-actin signaling.^111^ These findings suggest that SG modulation could help preserve chondrocyte function, although disease-stage specificity and safety require further validation.

#### Congenital skeletal disorders

Congenital skeletal disorders are characterized by bone fragility, recurrent fractures, progressive deformity, and multisystem complications that impair mobility and quality of life. Emerging evidence links LLPS and membraneless organelles to both chondrogenesis and osteogenesis. In chondrocytes, Sox9 recruits the 54-kD nuclear RNA-binding protein p54nrb to paraspeckles. p54nrb supports Sox9-mediated transcription of genes such as Col2a1 and, through its RNA-recognition motif, ensures proper splicing of newly transcribed Col2a1 pre-mRNA. Coupling transcription and splicing is essential for chondrocyte differentiation.^112^ Dysregulation of this mechanism is associated with skeletal dysplasias such as campomelic dysplasia, chondrodysplasia, and dwarfism. Rare-disease evidence further supports a condensate mechanism. In brachyphalangy-polydactyly-tibial aplasia syndrome, frameshift mutations in the C-terminal acidic tail of HMGB1 cause aberrant phase separation. Mutant HMGB1 accumulates in the nucleolar granular compartment, displaces NPM1, encases FIB1, disrupts nucleolar organization, impairs rRNA synthesis and protein translation, and induces cytotoxicity.^113^

**Fig. 5:**
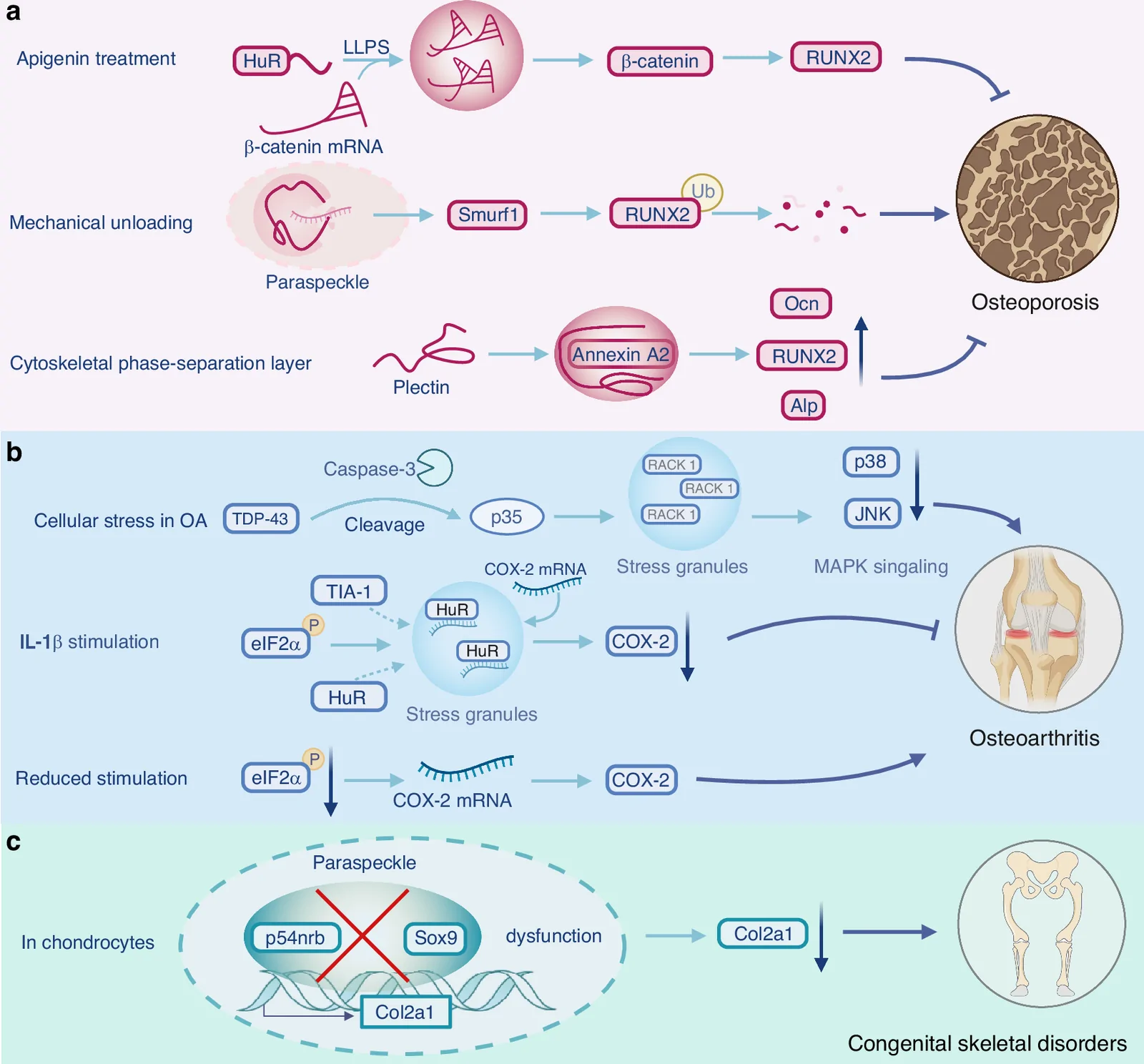
Roles of biomolecular condensates in bone diseases. **a** Osteoporosis. Apigenin treatment increases HuR abundance in senescent osteoblasts and promotes its condensation. HuR-containing condensates stabilize β-catenin mRNA and support the β‑catenin-RUNX2 signaling axis, alleviating osteoporotic progression. Conversely, mechanical unloading disrupts paraspeckle integrity, leading to Smurf1-mediated ubiquitination and degradation of RUNX2. At the cytoskeletal level, linker protein plectin undergoes phase separation to form condensates that sequester annexin A2, which is associated with upregulation of RUNX2 and osteogenic genes such as Ocn and Alp. **b** Osteoarthritis. Under OA-associated stress, a p35 fragment generated by caspase-3-mediated cleavage of TDP-43 promotes RACK1-dependent SG formation and modulates p38/JNK signaling, influencing inflammatory and apoptotic responses in chondrocytes. Upon IL-1β stimulation, HuR is recruited into SGs and binds COX-2 mRNA, suppressing its translation. Reduction in inflammatory stimuli leads to SG disassembly and restoration of COX-2 translation. **c** Chondrocyte function. Paraspeckle condensates recruit p54nrb and Sox9 to regulate Col2a1 transcription; dysregulation of this process is implicated in congenital skeletal disorders

Overall, condensates appear to participate in chondrocyte differentiation, transcriptional control, growth-factor signaling, and extracellular matrix regulation. Dysregulated condensates involving SOX9, RUNX2, RNA-binding proteins, or nucleolar components may contribute to impaired osteogenic or chondrogenic differentiation and to rare congenital bone phenotypes (Fig. 5). Systematic studies of condensate composition, dynamics, and functional consequences are still limited, leaving the molecular basis of LLPS-related skeletal abnormalities incompletely defined.

### Biomolecular condensates in bone tumors

Primary bone tumors and bone metastases disrupt bone architecture, compromise mechanical stability, and cause skeletal-related events such as pathological fracture, spinal cord compression, and bone pain.^114,115^ These complications markedly increase disability and mortality. Tumor invasion can also disrupt tendon-bone and ligament-bone interfaces, weakening load transfer across the musculoskeletal system. Because several bone tumors depend on transcriptional reprogramming, stress adaptation, and microenvironmental sensing, condensate biology provides a useful framework for understanding tumor progression and treatment resistance (Table 3).

**Table 3:** Biomolecular condensates in bone tumors

Disease	Subcellular localization	Core molecule	Recruited factors	Phenotype	Function
Osteosarcoma^120^	Nucleus	HOXB8, FOSL1	Pol II, BRD4, MED1	Tumor metastasis and chemoresistance	Drive the core regulatory circuit, promote the transcriptional elongation of oncogenes
Osteosarcoma^95^	Nucleus	L3MBTL2	Components of Polycomb repressive complex 1.6	Tumor suppression; inhibition of tumor growth and metastasis	Recruit PRC1.6 components and repress pro-oncogenic transcription
Ewing sarcoma^125^	Nucleus	ARID1A	BAF chromatin remodeling complex, EWSR1::FLI1	Enhance tumor cell proliferation, invasion, and migration; accelerate tumor growth in vivo	Create a chromatin remodeling hub that increases accessibility and activate oncogene transcription
Ewing sarcoma^126^	Nucleus	EWSR1::FLI1	BAF chromatin remodeling complex	Promote the proliferation, migration, and invasion of tumor cells.	Activate oncogenic transcriptional programs, remodel chromatin accessibility
Metastatic bone tumor^92^	Cytoplasm	DACT1	CK2	Increased colonization and growth of cancer cells in the bone, promote metastases	Sequester CK2 kinase and inhibit the Wnt/β-catenin signaling pathway
Metastatic bone tumor^133^	Cytoplasm	GRP78	Core chaperone proteins of endoplasmic reticulum stress	Maintain survival under hypoxia/ hypoglycemia stress	Pause global translation and reprogram the proteome
Metastatic bone tumor^135^	Cytoplasm	SYK	RNA-binding protein, autophagy-related factor	Promote breast cancer metastasis	P-bodies are cleared through autophagy, thereby promoting mesenchymal–epithelial transition

#### Osteosarcoma

Osteosarcoma, a common primary malignant bone tumor that predominantly affects children and adolescents, continues to be treated with standard chemotherapy regimens established in the 1970s, with no substantial improvement in survival rates over recent decades. The high degree of genetic heterogeneity and pathological complexity of osteosarcoma has led to repeated failures in clinical trials of targeted therapies and immunotherapies directed against oncogenic signaling pathways.^116,117^ More critically, the co-occurrence of lung metastasis and chemoresistance has become a central challenge in improving patient survival.^118^

In osteosarcoma, condensates can either suppress or promote tumor progression depending on the molecular scaffold involved. Ubiquitin-conjugating enzyme E2O binds the polybasic region of L3MBTL2 and mediates its polyubiquitination and degradation, causing loss of this tumor suppressor.^95,119^ Under physiological conditions, L3MBTL2 forms nuclear condensates that recruit Polycomb repressive complex 1.6 components and transcriptionally repress pro-oncogenic genes through its Pho-binding pocket. Arsenic trioxide inhibits UBE2O activity, stabilizes L3MBTL2, enhances L3MBTL2 condensate formation, and thereby restores tumor-suppressive activity.^95^ By contrast, oncogenic condensates can drive osteosarcoma metastasis and chemoresistance. HOXB8 and FOSL1 form IDR-mediated nuclear condensates at super-enhancers, increase chromatin accessibility, recruit RNA polymerase II, and activate metastasis- and drug resistance-associated genes. The H3K27 demethylase inhibitor GSK-J4 binds the HOXB8 IDR, disrupts its phase separation, and reverses invasive osteosarcoma phenotypes.^120^ WDR3 condensates also promote osteosarcoma progression, and nilotinib inhibits tumor growth by interfering with WDR3 condensate formation.^121^

The dual role of condensates in osteosarcoma pathogenesis, metastasis, and drug resistance is increasingly being uncovered, providing new insights into the molecular mechanisms of this disease. Current studies support the function of condensates in dynamically assembling transcriptional complexes with either tumor-suppressive or oncogenic roles and have begun to explore small-molecule interventions targeting disease-associated phase behavior. To translate these discoveries into clinical applications, several challenges must be addressed: first, most research has focused on transcriptional regulation, leaving the roles of condensates in post-translational modifications, cross-talk between signaling pathways, and remodeling of the tumor microenvironment poorly understood. Second, more clinically relevant models, such as patient-derived organoids or orthotopic metastasis models, are needed to validate condensate-targeted therapies. Advancing these efforts will help connect mechanistic studies of condensates to precision treatment strategies.

#### Ewing sarcoma

Ewing sarcoma is the second most common primary malignant bone tumor in children and adolescents. Its most characteristic molecular event is an oncogenic fusion protein resulting from a chromosomal translocation, making it a well-defined model for discussing condensate-driven oncogenic transcription. The most prevalent fusion type, EWSR1::FLI1, combines the low-complexity domains of the EWSR1 RNA-binding protein with the DNA-binding domain of the ETS transcription factor FLI1.^122,123^ The weak interactions between these two critical structural domains cooperatively promote LLPS of the fusion protein.

EWSR1::FLI1 forms intranuclear condensates through its N-terminal PrLD, where dense tyrosine residues act as molecular stickers. These dynamic condensates recruit the BAF chromatin-remodeling complex to GGAA microsatellite repeat elements and activate oncogenic transcription.^124,125^ ARID1A, the DNA-binding core subunit of the BAF complex, contains an N-terminal low-complexity/PrLD-like region that interacts with the EWSR1::FLI1 PrLD through tyrosine-tyrosine contacts. Cooperative phase separation of these PrLDs creates transcriptional factories that concentrate transcriptional machinery and chromatin remodelers.^125,126^ The FLI1 DNA-binding domain increases the rigidity of EWSR1-PrLD condensates through electrostatic interactions and promotes beta-sheet structures positive for Thioflavin T staining, a process linked to tumor-cell invasiveness. Conversely, DNA binding can limit interaction between the DNA-binding domain and the PrLD through steric hindrance, indicating reciprocal regulation between DNA binding and phase separation.^122^ Enrichment of ARID1A/EWSR1-FLI1 condensates in Ewing sarcoma patient tissues, but not normal tissues, further supports a tumor-specific role for phase separation.^125,127^ High-throughput screening identified abemaciclib (LY2835219) as a compound that dissolves these oncogenic condensates through the lysosomal pathway, reverses aberrant gene expression, and offers a therapeutic opportunity for Ewing sarcoma.^128^ Further work is needed to determine how condensate targeting affects chemoresistance, downstream transcriptional states, and immune-based therapies.

#### Metastatic bone tumor

During the progression of tumors, bones are among the most common sites of metastasis for many cancers, particularly breast and prostate cancer.^129^ The five-year survival rate for localized osteosarcoma can reach 70% with current combination therapies, whereas the five-year survival rate for metastatic osteosarcoma is considerably lower.^130,131^ During bone metastasis, tumor cells must adapt to the highly specialized bone marrow microenvironment, where hypoxia, low glucose availability, and abundant cytokines collectively form a complex regulatory network.^132^ Recent studies have revealed that LLPS of biomacromolecules plays a central role in the adaptability and plasticity of tumor cells. Particularly in multiple stages of bone metastasis, LLPS dynamically regulates key signaling pathways through the formation of membraneless organelles, thereby influencing tumor cell survival, phenotypic switching, and eventual colonization.

Biomolecular condensates mediate multiple aspects of cellular sensing within the bone microenvironment. Under the stressful conditions of hypoxia and low glucose in the bone marrow, disseminated tumor cells activate the unfolded protein response, notably upregulating the 78-kD glucose-regulated protein (GRP78), a UPR chaperone to form SGs, which maintain proteostasis and promote cell survival.^133^ These GRP78-positive condensates assemble during acute stress and disassemble upon adaptation, highlighting the dynamic regulatory role of condensates in response to microenvironmental pressures. TGF-β signaling plays a central role in breast cancer bone metastasis, supporting tumor cell colonization and progression.^134^ Shinde et al. showed that during TGF-β-induced epithelial-mesenchymal transition, SYK activity is substantially enhanced and localizes to P-bodies. SYK promotes autophagy-mediated clearance of LLPS-derived P-bodies, driving mesenchymal-epithelial transition and thereby supporting metastatic outgrowth. Inhibition of SYK activity or loss of the autophagy-related gene ATG7 prevents P-body clearance, impairs mesenchymal-epithelial transition, and suppresses metastasis.^135^ These findings indicate that the formation and clearance of LLPS-driven P-bodies are essential for regulating phenotypic plasticity. In terms of signal reprogramming, TGF-β signaling can also induce the expression of DACT1, which undergoes phase separation via its IDRs to form cytoplasmic biomolecular condensates. These DACT1 condensates do not colocalize with known organelles but sequester the Wnt pathway-activating kinase casein kinase 2, thereby inhibiting Wnt signaling and enhancing the bone-metastatic potential of breast and prostate cancer cells.^92^ This LLPS-mediated sequestration of casein kinase 2 provides a tunable mechanism for signaling insulation, which may be more effective than simple protein-protein inhibition alone.

**Fig. 6:**
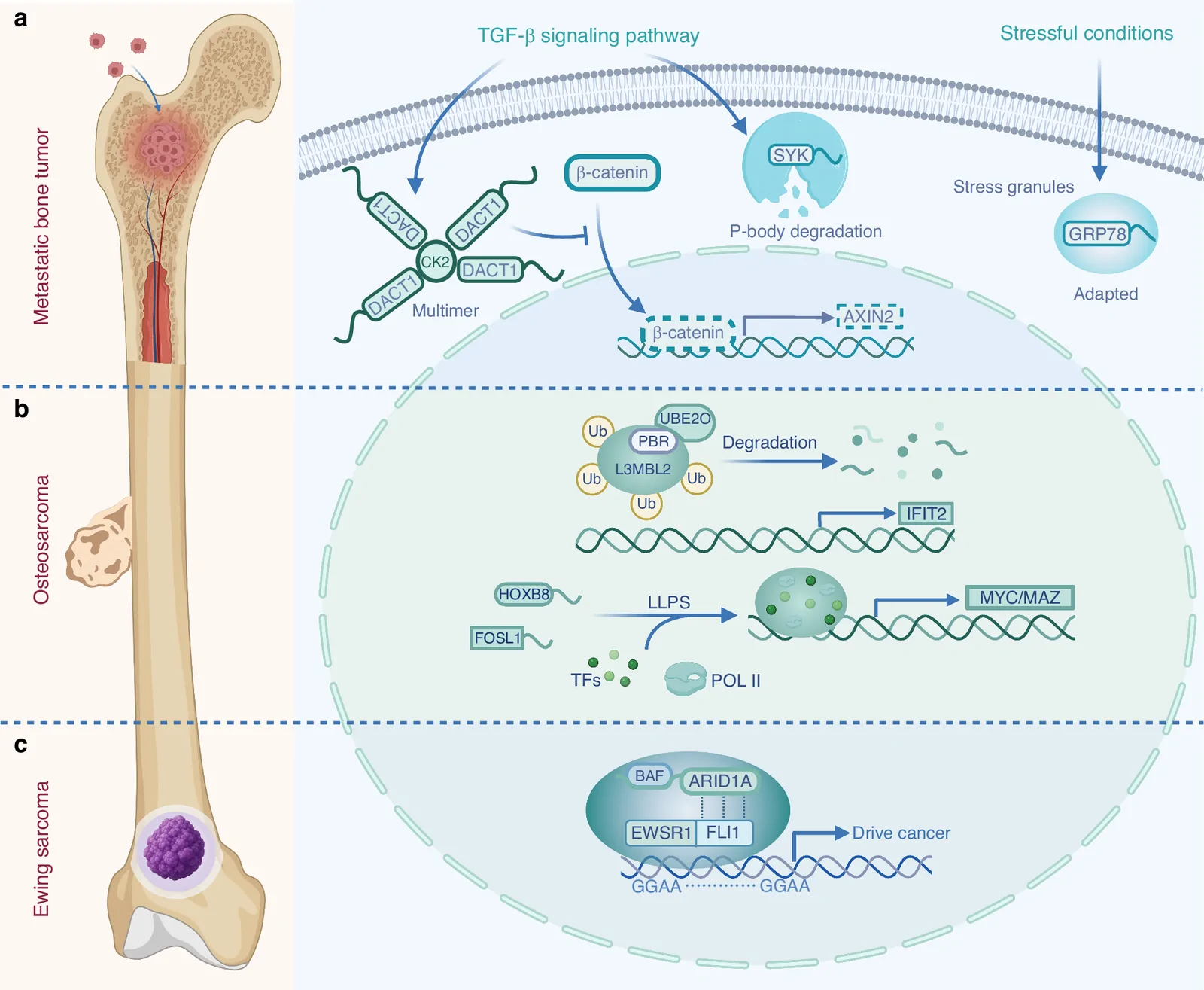
Roles of biomolecular condensates in bone tumors. **a** Adaptation to the bone marrow microenvironment. TGF-β signaling induces DACT1 expression, which undergoes IDR-mediated phase separation to form cytoplasmic condensates that suppress Wnt signaling by limiting β-catenin nuclear translocation—a process linked to enhanced metastatic traits. TGF-β also promotes SYK-mediated P-body degradation, supporting tumor growth. Under stress conditions such as hypoxia and low glucose, upregulated GRP78 assembles into survival-promoting SGs. **b** Transcriptional regulation in osteosarcoma. The tumor-suppressive L3MBTL2 condensate, which normally inhibits TNF/NF-κB signaling, is polyubiquitinated and degraded by the highly expressed E2 enzyme UBE2O. Conversely, condensates at super-enhancer regions increase chromatin accessibility and recruit RNA polymerase II, activating metastasis- and drug resistance–associated genes. **c** Oncogenic condensates in Ewing sarcoma. The EWSR1::FLI1 fusion protein forms condensates that recruit the BAF chromatin-remodeling complex to GGAA microsatellite repeats, driving oncogenic transcription. ARID1A acts as a molecular bridge via a shared N-terminal prion-like domain, stabilizing these transcriptional condensates

Studies of biomolecular condensates in bone tumors reveal their roles as regulatory assemblies that influence oncogenic transcription, cellular adaptation, and metastatic fitness (Fig. 6). In osteosarcoma, condensates show a dual role, with tumor-suppressive L3MBTL2 condensates and oncogenic HOXB8/FOSL1 hubs dynamically shaping tumor progression and chemoresistance. Ewing sarcoma is driven by the EWSR1::FLI1 fusion oncoprotein, whose PrLD-containing condensates act as neomorphic transcriptional factories that hijack chromatin remodeling to enforce a malignant state. Within the metastatic bone niche, condensates support tumor cell resilience through SG assembly, regulate phenotypic plasticity via clearance of P-bodies, and rewire signaling networks by sequestering key kinases. Future research must move beyond correlative observations to dissect the structural grammar of oncogenic condensates, clarify their interplay with the tumor microenvironment, and develop therapeutic approaches that precisely target the formation, composition, or dissolution of pathological assemblies.^136,137^

### Biomolecular condensates in soft tissue sarcomas

Soft tissue sarcomas can compromise musculoskeletal function, particularly when they arise in the limbs, trunk wall, or near joints and neurovascular structures.^138,139^ Recurrence near joints or other functional structures can cause severe limb dysfunction.^140^ The following sections review condensate-related mechanisms in soft tissue sarcomas, focusing on how LLPS-associated fusion oncoproteins remodel transcriptional programs and signaling pathways. This perspective may help identify vulnerabilities in treatment-resistant disease.

#### Low-grade fibromyxoid sarcoma

Low-grade fibromyxoid sarcoma is a rare malignancy that typically arises in deep soft tissues of the proximal extremities or trunk in young adults.^141^ Its rarity contributes to limited clinical experience and frequent diagnostic delay. More than 90% of cases carry a recurrent translocation that generates the FUS-CREB3L2 fusion oncoprotein.^142,143^ This chimeric protein contains canonical transmembrane domains and IDRs.^143^ IDRs within the FUS moiety drive LLPS at the endoplasmic reticulum membrane and initiate regulated intramembrane proteolysis, which releases the N-terminal fragment.^144^ After nuclear translocation, this fragment undergoes LLPS and assembles transcriptional condensates that concentrate coactivators, recruit chromatin remodelers, and activate a fibromyxoid sarcoma-specific gene-expression program.^144,145^ Although no targeted therapy is currently available, preclinical inhibition of fusion-oncoprotein nuclear translocation suppresses malignant phenotypes and may offer a therapeutic route.

#### Synovial sarcoma

Synovial sarcoma is another rare yet highly aggressive soft tissue sarcoma, accounting for 5%–10% of all soft tissue sarcomas.^146–148^ A major clinical concern is its metastatic propensity, and outcomes are poor once dissemination occurs.^149,150^ The oncogenic driver of synovial sarcoma is the SS18–SSX fusion gene.^151^ The QPGY domain of SS18, enriched in tyrosine residues, mediates LLPS and drives the formation of dynamic nuclear condensates. In parallel, SS18–SSX forms a stable heterodimer with BRG1, the catalytic subunit of the canonical BAF chromatin-remodeling complex, through its conserved SNH domain.^152^ LLPS-driven condensates may synergistically enrich BRG1, thereby enhancing local assembly in the crowded nuclear environment. SS18–SSX hijacks the BAF complex and redirects it to broad Polycomb domains, antagonizing PRC2-mediated silencing and activating bivalent developmental genes.^153^ Given the limited efficacy of current treatments, targeting phase separation offers a potential strategy to overcome therapeutic barriers in synovial sarcoma.^154^

#### Myxoid liposarcoma

Liposarcoma, the most common soft tissue sarcoma, is histologically classified into four major subtypes. Among these, the myxoid subtype accounts for approximately 30% of cases and is considered relatively chemosensitive compared with other liposarcoma subtypes. Its hallmark cytogenetic abnormality is the *t*(12;16) (q13;p11) translocation, which fuses DDIT3 (CHOP) at 12q13 with FUS at 16p11, generating the chimeric FUS-CHOP oncogene.^155^ This oncoprotein forms nuclear condensates that strongly colocalize with the super-enhancer marker BRD4 in myxoid liposarcoma cells and actively recruit BRD4 in heterologous systems, a spatial coupling that occurs independently of CHOP’s DNA-binding capacity.^156^ These findings reveal a dual-track mechanism: the FUS-derived PrLD orchestrates LLPS to generate a transcriptional hub, while the CHOP domain precisely anchors the complex to genomic target sites. Together, they establish a high local concentration of transcriptional regulators at super-enhancer regions, leading to aberrant activation of oncogenic gene networks. This mechanism clarifies how FUS-CHOP acquires its oncogenic potential and provides a compelling therapeutic rationale for targeting the condensate assembly process in myxoid liposarcoma.

Evidence from fibromyxoid sarcoma, synovial sarcoma, and myxoid liposarcoma supports a shared paradigm in which oncogenic fusion proteins exploit condensate formation to remodel transcriptional programs and chromatin landscapes, thereby driving tumor initiation, progression, and recurrence. The IDRs and PrLDs embedded in these fusion proteins act as molecular scaffolds to nucleate condensates, enrich transcriptional machinery, and rewire epigenetic regulation. While the precise in vivo dynamics of these condensates remain incompletely understood, accumulating findings support their central role in malignant phenotypes and treatment resistance. Targeting condensate formation or downstream condensate-dependent transcriptional machinery thus represents a possible therapeutic route for soft tissue sarcomas.

### Biomolecular condensates in skeletal muscle disorders

Skeletal muscle disorders directly impair musculoskeletal structure and function. Clinically, they reduce contractile strength, coordination, limb function, ambulation, and, in severe cases, respiratory capacity.^157^ Muscle dysfunction can also aggravate osteoarthritis, osteoporosis, and intervertebral disc degeneration by altering joint loading, increasing fibrosis, impairing bone regeneration, and sustaining inflammation.^158,159^ The following sections focus on condensate mechanisms that are directly relevant to skeletal muscle development, degeneration, repair, and inherited myopathies (Table 4).

**Table 4:** Biomolecular condensates in skeletal muscle disorders

Disease	Subcellular localization	Core molecule	Recruited factors	Phenotype	Function
Muscular dystrophy^26^	Cellular membrane, cytoplasmic	PrPC	miR-214-3p/miR-204-5p	Autophagy inhibition, abnormal muscle bundle formation	Selectively recruit miRNAs, inhibit ATG5 translation, and impair autophagy pathways
Muscular dystrophy^172^	Nucleus	GYS1	RNA Pol II, MyoD	Blocked differentiation, impaired regeneration and repair	Inhibits glycogen synthesis, drives myogenic gene expression
Muscular dystrophy^47^	Nucleus	hnRNPDL	TNPO1, SRSF1	Increase protein insolubility and myofibrillar disorganization	Involved in mRNA processing and the regulation of alternative splicing
Muscle atrophy^67^	Nucleus	SUZ12	Components of Polycomb repressive complex 2	Intramuscular fat deposition, mitochondrial dysfunction	Catalyzes H3K27me3, repressing the expression of Fhl3 and Rnf128
Muscle atrophy^66^	Nucleus	MEIS2	Transforming growth factor-β receptor II promoter	Delay muscle regeneration	Inhibit the expression of TGF-β/SMAD3 pathway
Muscle atrophy^90^	Cytoplasm	YTHDF2	TM4SF1 mRNA	Muscle differentiation is hindered	Induce degradation of TM4SF1 mRNA
Muscle atrophy^163^	Nucleus	RPS27L	IGF1	Muscle mass and cross-sectional area of muscle fibers have significantly increased	Activate the downstream signaling pathways
Muscle atrophy^89^	Nucleus, cytoplasm	MEF2D	Myogenic regulatory factors	Promote the formation of multinucleated myotubes in the early stages of differentiation and accelerate the differentiation process	Regulate the transcription of genes related to muscle differentiation and metabolism
Muscle atrophy^162^	Nucleus	PGC-1α	Pol II, H3K27ac	Alteration in basal oxygen consumption of skeletal muscle	Assemble transcriptional hubs to efficiently coordinate the transcriptional processes of metabolism-related gene programs
Muscle atrophy^85^	Nucleus	TAZ	Smad7, β-catenin	Impairment of myotube formation	Assemble inhibitory complexes to repress the transcription of muscle differentiation-related genes
Muscle atrophy^94^	Cell membrane	FGF	FGFR	Characterized by faster clearance of necrotic areas	Condensates concentrate FGF and overcome the signal activation threshold.
Distinct hereditary myopathy^174^	Cytoplasm	TIA1	Ubiquitin conjugates, SQSTM1	Presence of rimmed vacuoles and protein aggregates within muscle fibers	Leading to the accumulation of toxic proteins and cell death
Distinct hereditary myopathy^173^	Cytoplasm	TDP-43	Autophagy-related protein	Myasthenia, vacuolar degeneration	Condensates cause abnormal retention of TDP-43 in the cytoplasm

#### Muscle atrophy

Muscle atrophy causes progressive loss of muscle mass and function, limiting mobility and quality of life. Its mechanisms remain complex and incompletely treatable. Skeletal muscle development requires myoblast proliferation, differentiation, myotube fusion, and maturation into myofibers.^160^ Myogenic regulatory factors, including Myf5, MyoD, myogenin, and MRF4, coordinate this process.^161^ Condensates help organize the transcriptional and post-transcriptional programs that support myogenesis. MEF2D tunes its own phase separation to coordinate with basal transcription machinery and myogenic factors, enabling lineage specification.^89^ PGC-1α forms LLPS-driven nuclear condensates that regulate metabolism-related genes and may be therapeutically relevant in disuse atrophy.^162^ lncRNAs such as MEG3 and lnc-RMG indirectly regulate muscle-development genes by modulating LLPS-linked pathways.^66,67^ TAZ, a Hippo pathway effector, forms phase-separated nuclear condensates and represses muscle-specific gene expression through interactions with Smad7 and beta-catenin.^85^ At the post-transcriptional level, METTL3-mediated m^6^A modification of TM4SF1 mRNA promotes recognition by YTHDF2 condensates, leading to mRNA degradation that limits proliferation and supports differentiation.^90^ Rps27l also undergoes LLPS and interacts with IGF1 to regulate myogenesis, although its downstream pathways remain unclear.^163^

Condensate-dependent nuclear bodies, including paraspeckles and nuclear speckles, act as hubs for RNA metabolism during myogenesis.^164,165^ During C2C12 myoblast differentiation, transcription of the MEN epsilon/beta locus increases, producing two nuclear isoforms that localize to paraspeckles and scaffold core proteins such as NONO.^166^ DGK-ζ, an intranuclear lipid kinase, regulates myogenic differentiation by localizing to nuclear speckles and the nuclear matrix, interacting with signaling molecules such as phosphoinositide-specific phospholipase C beta 1, and tuning the nuclear DG/PA balance.^167^ LLPS also contributes to intercellular communication. The macrophage-derived metabolite 11,12-epoxyeicosatrienoic acid is released through Gasdermin D pores and enhances FGF signaling by promoting fibroblast growth factor phase separation.^94^ This response supports tissue repair and mitigates age-associated decline in regeneration. Together, these mechanisms show how condensates integrate transcription, RNA processing, metabolism, and intercellular signals during muscle development and atrophy.

#### Muscular dystrophy

Muscular dystrophies are severe, progressive diseases that lead to muscle degeneration, loss of mobility, and early mortality. Despite genetic insights, effective treatments remain scarce, as the molecular mechanisms driving these conditions are not fully understood.^168^ Accumulating evidence suggests that several forms of muscular dystrophy share a core pathological pathway driven by abnormal LLPS mediated by RNA-binding proteins.^169^ The central mechanism of this pathway involves the disruption of RNA processing homeostasis within the nucleus, which brings distinct pathogenic factors, such as structural mutations, splicing abnormalities, and metabolic stress, into a common phenotype of impaired myogenesis and muscle fiber degeneration. In limb-girdle muscular dystrophy type 1 G, pathogenic mutations in hnRNPDL or alterations in its alternative splicing isoforms disrupt the protein’s intrinsic phase separation properties, supporting the transition of reversible condensates into insoluble amyloid-like aggregates. This aberrant phase transition promotes hnRNPDL insolubility and functional impairment, potentially leading to dysregulation of RNA processing and ultimately contributing to progressive muscle fiber degeneration.^47^ Paraspeckles and nuclear speckles have been implicated in oculopharyngeal muscular dystrophy.^170,171^ Alanine-expanded PABPN1 accumulates at speckle peripheries to form intranuclear inclusions, leading to PABPN1 and poly(A) RNA depletion, thereby disrupting mRNA processing and transport.^171^ PABPN1 also interacts with the nuclear matrix protein MATR3 to regulate selective polyadenylation, intron retention, and stability of the long noncoding RNA NEAT1.^170^ NEAT1 serves as a paraspeckle scaffold, and its accumulation increases paraspeckle abundance and drives aberrant RNA editing.^164^ Condensate dysregulation not only affects RNA metabolism but also incorporates metabolic regulation into the dystrophic network. GYS1 and NONO/p54nrb form nuclear condensates that co-assemble with MyoD and the transcription initiation complex to regulate the expression of myogenic genes such as myogenin and Tnni2. Disruption of this system leads to pathological glycogen accumulation, mitochondrial dysfunction, and impaired muscle regeneration.^172^ Abnormal PrPᶜ condensates specifically bind miR-214-3p, inhibiting the translation of the autophagy gene ATG5, thereby exacerbating muscle cell death and regeneration defects.^26^ Overall, these studies establish condensate dysfunction as a unifying pathogenic axis in muscular dystrophies: various genetic or stress-induced factors can convert physiological condensates into cytotoxic aggregates, thereby compromising proteostasis, transcriptional fidelity, and cellular resilience.

#### Distinct hereditary myopathy

Recent studies on hereditary myopathies highlight that mutations in LCD-containing RNA-binding proteins perturb LLPS behavior and converge on a shared pathway of dysregulated stress-granule homeostasis and impaired proteostasis in skeletal muscle. A representative example is the newly identified G376V variant of TDP-43, which resides in its prion-like LCD and markedly enhances cytoplasmic condensate formation, promotes assembly into higher-order oligomers, and alters condensate morphology. These aberrant phase-separated assemblies accumulate in patient-derived myoblasts and are associated with cryptic exon inclusion and myofibrillar disorganization, ultimately producing a primary muscle-intrinsic degenerative phenotype distinct from ALS.^173^ Similarly, pathogenic variants in TIA1, another LCD-containing SG nucleator, such as the N357S allele, shift the phase diagram toward enhanced LLPS and markedly delay SG disassembly. The TIA1-N357S variant synergizes with SQSTM1 mutations to drive pathology: the former impairs SG dynamics by enhancing LLPS, while the latter compromises protein clearance capacity by disrupting autophagic flux. Together, they lead to the accumulation of ubiquitinated proteins within persistent SGs, which gradually evolve into amyloid-like, rimmed vacuole-associated inclusions characteristic of multisystem proteinopathy.^174^ These findings delineate a unifying molecular axis in which LCD mutations increase condensate stability and decrease SG dynamics, thereby overwhelming ubiquitin-dependent autophagic clearance. The resulting persistence and pathological hardening of phase-separated RNP assemblies form a mechanistic continuum that explains how distinct genetic lesions, TDP-43 G376V monogenic myopathy versus TIA1-SQSTM1 digenic MSP, produce phenotypically varied yet mechanistically convergent muscle atrophy.

**Fig. 7:**
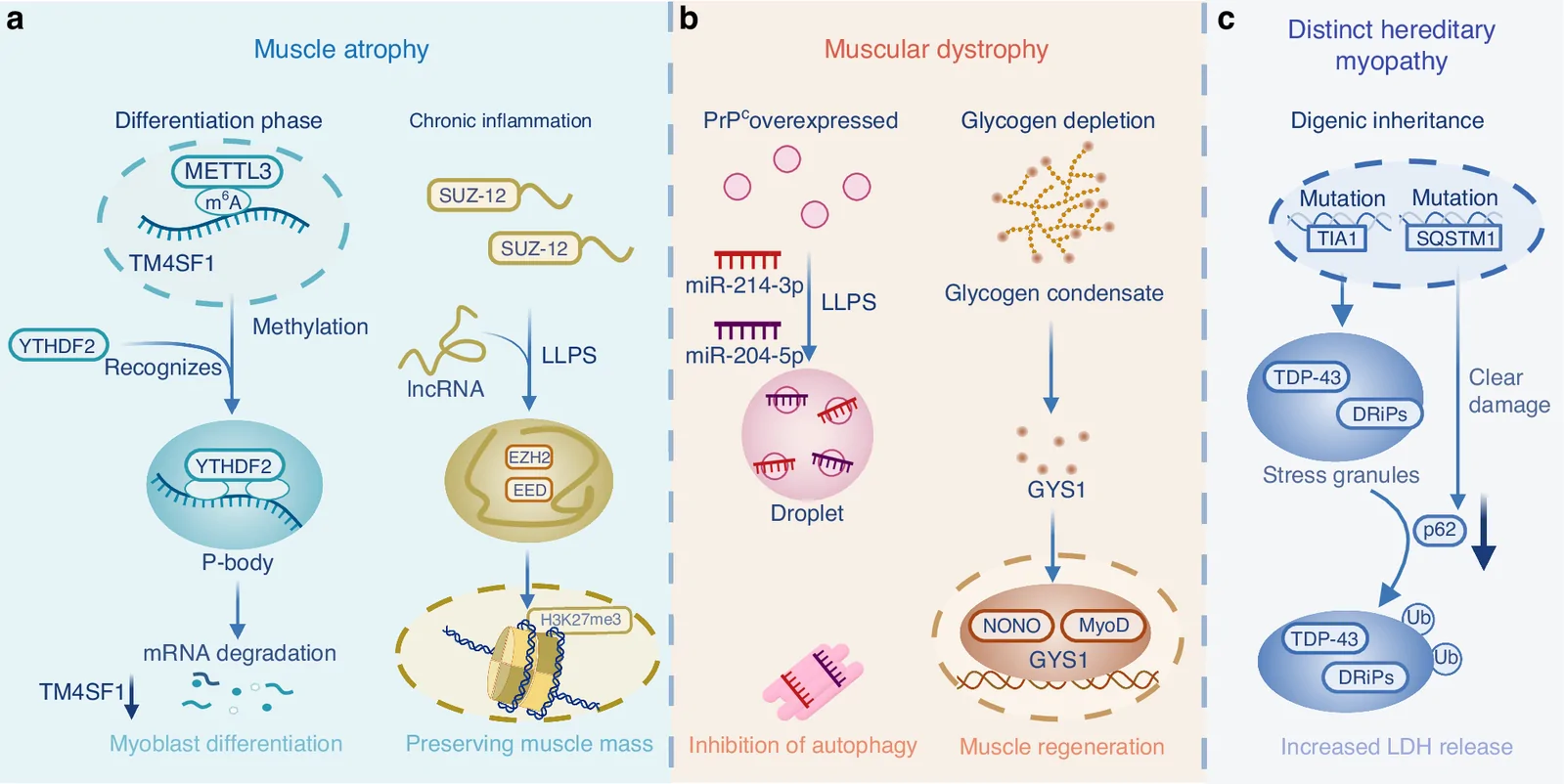
Roles of biomolecular condensates in skeletal muscle disorders. **a** Muscle atrophy. During myoblast differentiation, METTL3-mediated m⁶A modification of TM4SF1 mRNA supports its recognition by YTHDF2-containing condensates, promoting mRNA decay via P-bodies and supporting myogenic differentiation. Under chronic inflammation, lncRNA-associated phase separation recruits Polycomb repressive complex components, leading to histone modifications and indirect regulation of muscle-related gene expression that contributes to muscle mass maintenance. **b** Muscular dystrophy. Overexpressed cellular prion protein (PrPᶜ) recruits miR-214-3p/miR-204-5p family members into phase-separated condensates that can mature into fibrillar assemblies, associated with autophagy inhibition, muscle cell death, and impaired regeneration. Under glycogen depletion, glycogen synthase 1 translocates to the nucleus and forms condensates that interact with myogenic regulators, modulating transcription of myogenesis-related genes and supporting context-specific muscle regeneration. **c** Hereditary myopathy. In digenic inheritance-associated myopathy, TIA1 mutations enhance SG assembly, whereas p62 mutations impair their clearance. This leads to persistent SGs enriched in TDP-43 and defective ribosomal products, resulting in increased cytotoxicity and elevated LDH release

Across muscle atrophy, muscular dystrophies, and hereditary myopathies, condensate dysregulation has emerged as a mechanism that integrates diverse pathogenic drivers into a shared trajectory of muscle fiber degeneration (Fig. 7). Under physiological conditions, condensates orchestrate myogenic transcriptional programs, RNA metabolism, and metabolic signaling; however, mutations, cellular stress, or aging can shift these assemblies toward pathological hardening, thereby disrupting RNA homeostasis, proteostasis, and muscle regeneration. Despite this progress, major gaps remain. Most evidence comes from in vitro models, leaving the in vivo behavior of condensates in mature or diseased muscle insufficiently understood. Direct causal links between condensate dysfunction and atrophy require more rigorous biophysical and genetic validation. The upstream signals that regulate condensate dynamics and the molecular composition of these structures remain poorly defined. Addressing these limitations will be important for advancing condensate-based therapeutic strategies for muscle atrophy and myopathies.

### Biomolecular condensates in neuromusculoskeletal disorders

The motor system functions as an integrated unit in which the nervous system and musculoskeletal apparatus are tightly coupled. This connection justifies considering selected motor-system disorders when their neurological pathology substantially impairs musculoskeletal performance. The neuromuscular junction provides a clear example. During synapse formation, Rapsyn undergoes LLPS and creates dynamic condensates that concentrate acetylcholine receptors, anchoring proteins, and regulatory signals.^93,175^ These membraneless compartments support acetylcholine receptor clustering and synaptic maturation.^93^ Importantly, congenital myasthenic syndrome mutations in Rapsyn disrupt its phase-separation capacity, directly linking condensate dysfunction to impaired neuromuscular transmission. The neuromuscular junction, therefore, illustrates how LLPS can connect neuronal signaling to musculoskeletal performance.

#### Amyotrophic lateral sclerosis

ALS is a fatal neurodegenerative disorder characterized by progressive motor-neuron loss, muscle atrophy, paralysis, and respiratory failure. Average survival after diagnosis is typically 2–5 years, and no curative treatment is available.^176^ Aberrant condensate assembly is closely linked to ALS pathogenesis, with TDP-43 aggregation into irreversible amyloid fibrils recognized as a key pathological mechanism. These aggregates sequester normal TDP-43 and disrupt essential cellular processes, causing proteotoxic stress and neuronal death.^177^ Recent studies have clarified how TDP-43 aggregation occurs. TDP-43 first concentrates within SGs beyond a critical threshold, and oxidative stress then triggers intra-droplet phase separation from RNA and G3BP1, producing a TDP-43-enriched liquid phase. This phase undergoes liquid-to-solid transition as low-complexity domains shift from alpha-helical to beta-sheet conformations, eventually forming insoluble aggregates with pathological phosphorylation and ubiquitination.^178^ This mechanism has been validated in cellular models, mouse models, and patient samples. External factors can further accelerate pathology. Nanoscale polystyrene particles induce oxidative stress, retain TDP-43 in the cytoplasm, and promote phosphorylated pathological aggregates that cause neuronal injury and ALS-like behavioral deficits in mice.^179^ Hyperosmotic stress similarly favors TDP-43 liquid-to-solid transition when protective factors are absent, suggesting that enhancing condensate dynamics may be therapeutically useful.^180^

#### Huntington’s disease

Huntington’s disease is an inherited neurodegenerative disorder characterized by choreiform movements, cognitive decline, and neuropsychiatric symptoms.^181^ Its motor manifestations directly affect gait, coordination, and skeletal muscle use, which makes the disease relevant to neuromusculoskeletal dysfunction. Mutant huntingtin impairs autophagosome formation by disrupting the phase-separation properties of SQSTM1 droplets and altering phagophore membrane curvature. This defect promotes toxic protein accumulation, energy depletion, and oxidative stress.^182^ Although the primary pathology is neuronal, condensate dysfunction may therefore worsen motor-system performance by disturbing autophagy and proteostasis at the neuron-muscle interface. These findings support a focused, rather than general, role for condensates in Huntington’s disease.

#### Parkinson’s disease

Parkinson’s disease is a rapidly growing neurodegenerative disorder whose cardinal motor features arise from degeneration of dopaminergic neurons in the substantia nigra pars compacta.^183–185^ These motor deficits directly impair posture, gait, coordination, and skeletal muscle control. A key molecular event is α-synuclein aggregation, which can begin when α-synuclein undergoes LLPS to form liquid-like droplets. These droplets can mature through liquid-to-solid transition into beta-sheet-rich oligomers, fibrils, and hydrogel-like matrices that stabilize pathological aggregates.^186^ De novo macrocyclic peptides can induce α-synuclein LLPS by interacting mainly with its C-terminal region and altering droplet maturation.^187^ Conversely, β-synuclein co-condenses with α-synuclein, reduces protein mobility within condensates, blocks droplet fusion, and suppresses amyloid maturation.^188^ Thus, condensate-mediated alpha-synuclein assembly is relevant to Parkinsonian motor dysfunction, but its musculoskeletal relevance is indirect and mediated through neuronal control of movement.^189,190^

Aberrant condensate assembly has emerged as a pathophysiological mechanism in diverse musculoskeletal disorders. Evidence from osteoporosis, osteoarthritis, skeletal developmental anomalies, bone and soft tissue tumors, and muscle or injury-related conditions suggests that condensates govern cellular events such as chromatin organization, transcriptional regulation, stress responses, and signaling dynamics. Dysregulated condensates can disrupt the balance between bone formation and resorption, contribute to aberrant chondrocyte homeostasis, support malignant transformation, and impair muscle regeneration. Biomolecular condensates also provide a framework linking genetic mutations, epigenetic alterations, and environmental stressors to disease onset and progression. These insights expand musculoskeletal pathobiology beyond traditional linear signaling pathways and identify pathological condensates as possible therapeutic targets. Future studies should focus on the spatiotemporal regulation of condensates, their cross-talk with mechanical and metabolic cues, and their druggability.

## Clinical translation of biomolecular condensate biology

### Prognostic model development

Condensate biology may also inform prognosis and therapy guidance in musculoskeletal disease. Rather than measuring molecular abundance alone, condensate-based biomarkers could capture functional states such as scaffold recruitment, material hardening, stress-granule persistence, or oncogenic transcriptional hub formation.^191–194^ Existing LLPS-related risk models outside the musculoskeletal field, including esophageal adenocarcinoma, provide methodological proof of principle but are not direct disease analogs.^195^ In musculoskeletal oncology, multi-omics data from osteosarcoma and related tumors could be used to build condensate-related prognostic models that predict survival, reflect tumor microenvironmental states, and nominate drug sensitivities. However, direct biomarker studies across osteoporosis, OA, muscle disease, and neuromuscular disorders remain limited. Future models should therefore be anchored to musculoskeletal cohorts and validated against condensate-specific biology rather than using generic LLPS gene lists alone.^121,195^

### Targeting pathological condensates

As described above, aberrant condensate assembly or maturation can contribute to the pathogenesis and progression of several musculoskeletal disorders. Therapeutic strategies that target disease-associated condensates, therefore, represent a possible route for treating these diseases, provided that physiological condensates are preserved. This approach has gained traction in oncology, where interventions that disrupt IDRs or alter condensate dynamics have shown efficacy. For example, Xie et al. developed a series of FOXM1-interfering peptides based on its IDR1 structure; among these, FIP4, which contains a phosphomimetic S376 residue, effectively disrupts FOXM1 phase separation, suppressing tumor cell proliferation, migration, and invasion while enhancing immunogenic gene expression.^196^ Similarly, SI-2 binds directly to the IDR of SRC-3, blocking its interaction with NSD2 and disrupting the SRC-3/NSD2 complex in multiple myeloma, thereby resensitizing cells to bortezomib and promoting apoptosis.^197^ Beyond conventional small-molecule inhibitors, innovative condensate-targeting modalities continue to emerge. For instance, a near-infrared light-controlled system employing upconversion nanoparticles can induce DNA phase separation and generate singlet oxygen, synergistically damaging glioma cell DNA and suppressing tumor growth.^198^ Alternatively, peptide-based coacervates formed via phase separation have been used to engineer immune cell surfaces for enhanced antibody display and targeted cancer cell killing.^199^

Some compounds can also promote phase separation for therapeutic benefit. icFSP1 induces FSP1 phase separation, suppresses its anti-ferroptosis activity, and strongly potentiates ferroptosis under GPX4 inhibition, suggesting an anticancer strategy.^200^ By contrast, the conventional enzymatic inhibitor iFSP1 does not overcome resistance linked to aberrant protein localization, illustrating why the condensate state can matter beyond catalytic activity.^200,201^ In skeletal tumors, GSK-J4 disrupts HOXB8/FOSL1 condensates and blocks super-enhancer-driven oncogenic transcription in metastatic or therapy-resistant osteosarcoma.^120^ Nilotinib interferes with WDR3 phase separation and modulates the RASSF1A-Hippo pathway, spliceosome function, lysosomal degradation, and immune-microenvironment features to inhibit osteosarcoma progression.^121^ High-throughput screening and imaging-based platforms further support the discovery of condensate-modulating molecules.^128,202^

Translation of musculoskeletal disease faces several barriers. First, target definition is difficult because physiological condensates support normal transcription, RNA metabolism, DNA repair, and stress responses. Selectivity will likely require disease-enriched scaffolds, mutation-dependent condensates, or pathological material states such as persistent, hardened, or mislocalized assemblies. A second strategy is to target condensate-specific clients, post-translational modifications, or upstream signals rather than the phase transition itself. Local delivery may further reduce toxicity for intra-articular, intratumoral, or biomaterial-based applications. Tissue access is another barrier: mineralized bone, avascular cartilage, fibrotic muscle, and the neuromuscular junction impose different diffusion, retention, and clearance constraints. Finally, condensate modulators may have narrow dose windows, context-dependent effects, and uncertain pharmacokinetics because condensate partitioning can alter local drug concentration. Therapeutic studies should therefore measure condensate behavior, downstream function, tissue distribution, and off-target effects in parallel.

### Condensate-inspired delivery systems

Therapeutic biomacromolecules hold great promise for treating a wide spectrum of diseases; however, their clinical translation has long been hampered by inefficient intracellular delivery. Condensate-inspired strategies provide a new means of overcoming this bottleneck, offering distinct advantages over conventional delivery methods. For instance, histones and Chol-ssDNA can undergo LLPS at defined ratios and concentrations to form core–shell condensates that efficiently encapsulate negatively charged drugs via electrostatic interactions. These complexes are internalized through a receptor-independent macropinocytosis pathway, while simultaneously providing protective shielding and prolonged in vivo retention, thereby markedly enhancing drug delivery efficiency and therapeutic efficacy.^203^ Similarly, early studies showed that LLPS-driven conjugated peptides readily traverse cellular membranes and rapidly recruit diverse macromolecules.^204^ Building on this, subsequent work employed modular design of phase-separating peptides to tune the physicochemical properties of coacervate microdroplets, resulting in a universal and efficient platform with programmable release kinetics for intracellular macromolecule delivery.^205^

Traditional delivery systems often trap proteins in endosomes and prevent efficient cytoplasmic release. By contrast, the light-responsive phase-separating fluorescent molecule PPFM undergoes Norrish type II photolysis after 405 nm irradiation, triggering condensate disassembly and protein release.^206^ Phase separation can also be used at the cell membrane to enhance drug enrichment and uptake. One system anchors a targeting component to cancer-cell receptors and initiates in situ co-phase separation of a drug-conjugated partner. The resulting condensates create high local drug concentration, enter cells through clathrin-independent endocytosis, and release cytotoxic drugs after lysosomal acidification.^207^ Another approach uses alkaline phosphatase-catalyzed dephosphorylation and self-assembly of the phosphopeptide KYp to induce lipid-protein phase separation at the cell membrane, increasing membrane permeability and peptide-drug internalization.^208^ These strategies show how LLPS can support electrostatic assembly, enzyme-triggered self-assembly, drug enrichment, and controlled uptake.

Condensate-inspired delivery platforms may be useful for musculoskeletal disorders, but in vivo translation remains difficult. In OA, therapeutic efficacy is limited by the dense, negatively charged, and avascular cartilage matrix, which restricts particle penetration, and by rapid clearance through synovial fluid turnover.^209,210^ A system that works in monolayer chondrocyte culture may fail in intact cartilage because of size exclusion, electrostatic trapping, enzymatic degradation, and nonuniform loading during joint movement. Dense tendon and mineralized bone pose different barriers, including poor vascular access, fibrotic scarring, mineral binding, and slow convective transport.^211^ Safety is also unresolved. Phase-separating carriers may alter local protein condensation, inflammatory signaling, or extracellular matrix organization, especially after repeated dosing. Future studies should therefore move from proof-of-concept uptake assays to explants, mechanically loaded cartilage or tendon models, large-animal joints, and disease models that measure tissue penetration, residence time, release kinetics, immune activation, and functional recovery.

### Hydrogel-induced modulation of intracellular condensates

The physical properties of the extracellular environment, especially matrix stiffness, can regulate intracellular condensates and provide a materials-based route to modulate cell behavior. YAP, a key mechanosensing transcriptional coactivator, undergoes LLPS and forms condensates in response to stiff extracellular matrices.^212,213^ Hydrogels can exploit this principle by tuning mechanical cues that influence condensate formation. Reprogramming-responsive hydrogels with autonomous stiffness changes trigger YAP condensate formation at key stages of cellular reprogramming and accelerate induction of pluripotent stem cells.^214^ Similarly, hydrogel elastic modulus regulates transcription-factor recruitment through YAP-mediated LLPS and promotes osteogenic differentiation of human bone marrow stromal cells, suggesting relevance for bone regeneration.^215^

These findings suggest a material-condensate-cell fate axis. Instead of directly targeting proteins with chemical agents, smart biomaterials may manipulate intracellular condensates by controlling matrix mechanics. Applications to muscular dystrophy, tendon repair, cartilage regeneration, and arthritis remain early. Future studies should test how defined mechanical signals regulate condensates in musculoskeletal cells and whether correcting condensate behavior improves tissue repair.

## Challenges and prospects

### Human-relevant musculoskeletal models for clinical translation

Muscle tissue contains multiple cell types, and current isolation methods can introduce non-target-cell contamination, reducing the purity and yield of populations such as muscle stem cells. These cells are difficult to maintain in a quiescent state in vitro and often activate, differentiate, or undergo apoptosis, leading to loss of regenerative potential. As a result, many studies still rely on animal models. This creates a translational gap because condensate behavior observed in animal or simplified culture systems may not reflect human clinical tissue.

More physiologically relevant human models are needed, but they must be designed around the specific questions that condensate studies ask. Muscle organoids, 3D myotube cultures, patient-derived induced pluripotent stem cells, bone-on-chip systems, cartilage explants, and osteoblast-osteoclast-immune co-cultures can better preserve cell-cell interactions, extracellular matrix stiffness, nutrient gradients, and mechanical loading than conventional two-dimensional cultures. Each model also has limits. Organoids may lack vascularization, innervation, immune components, and mature force generation; 3D cultures can be difficult to image at subcellular resolution; and patient-derived systems vary with donor age, disease stage, medication exposure, and genetic background. For condensate research, model validation should include not only lineage markers and tissue morphology, but also condensate-specific readouts such as endogenous localization, material properties, FRAP recovery, stress responsiveness, and rescue by phase-deficient mutants. When these models are combined with single-cell RNA sequencing, spatial transcriptomics, proteomics, and well-annotated clinical biobanks, they can help determine whether condensates observed in vitro also exist in diseased human tissue and whether they drive pathology rather than simply accompany it.

### Live imaging and mechanical regulation of condensates

Tissues such as muscle, bone, cartilage, and the neuromuscular junction are mechanically active and structurally complex. Several mechanosensitive condensate regulators are particularly relevant to human musculoskeletal tissues. YAP and TAZ are key mechanosensitive transcriptional regulators that respond to matrix stiffness and cytoskeletal tension. Emerging studies further suggest that TAZ or YAP-associated condensates can translate mechanical cues into transcriptional outputs.^85,212,213^ NEAT1-dependent paraspeckles respond to mechanical loading in osteoblasts and control Smurf1 mRNA retention, thereby affecting RUNX2 stability.^97^ RUNX2 itself forms condensate-like assemblies that connect non-coding variants to osteogenic gene regulation.^88^ In Drosophila muscle, Tono senses compressive forces generated during myofibril and mitochondrial growth and forms nucleoplasmic droplets that regulate developmental transcription.^84^ Osmotic and metabolic changes also feed into mechanosensitive condensate biology; mechano-dependent sorbitol accumulation can support condensate formation, suggesting that load may regulate condensates through both force transmission and intracellular solute balance.^212^ Altered loading in osteoporosis, sarcopenia, disuse, or muscle injury may therefore change condensate thresholds, residence time, or client recruitment. Real-time, high-resolution observation of these events in vivo remains difficult, and causal links between condensate behavior and outcomes such as bone strength or muscle contractility remain incomplete.

To address these limitations, future studies should combine condensate-sensitive biosensors, endogenous fluorescent tagging, optogenetic control of condensate assembly, and super-resolution live-cell imaging. Mechanical stimulation platforms should be matched to tissue context: cyclic stretch for muscle, compression for cartilage, fluid shear or substrate stiffness gradients for bone cells, and engineered neuromuscular junction systems for synaptic organization. AI-assisted image analysis and physical modeling can help quantify condensate number, size, fusion, dissolution, and molecular exchange under defined loading regimens. These approaches would make it possible to test whether altered mechanical load changes condensates directly, whether the effect is mediated through YAP/TAZ, NEAT1, RUNX2, Tono, osmotic adaptation, or metabolic pathways, and whether correcting condensate behavior restores musculoskeletal function.

### Physical properties and functions of condensates

The physical properties of musculoskeletal condensates, including viscoelasticity, interfacial tension, and stiffness, remain poorly understood, especially in mechanosensing and spatiotemporal regulation.^216^ Microfluidic devices can model fluid shear stress, stretch systems can impose cyclic strain, and atomic force microscopy and microrheology-based approaches can help quantify condensate or condensate-associated mechanics. Fluorescence correlation spectroscopy can measure molecular dynamics, while molecular dynamics simulations can clarify assembly and dissolution at the molecular level. Combining these approaches may establish causal links between mechanical stimulation, condensate material properties, and cellular function. Such knowledge should improve understanding of muscle atrophy, osteoporosis, arthritis, and related disorders and guide more selective therapeutic strategies.

## Conclusion

Biomolecular condensates are an organizing principle in the musculoskeletal system, but their disease relevance varies by context and by evidence strength. Dynamic condensates regulate gene expression, signal transduction, stress adaptation, and tissue repair. When their composition, material state, localization, or clearance is altered, they may contribute to congenital skeletal abnormalities, muscle atrophy, osteosarcoma, metastatic bone disease, and neuromuscular disorders. In several settings, however, condensates may also represent adaptive responses to stress rather than primary disease drivers. Progress will depend on experiments that separate direct causality from association, define disease-specific condensate features, and validate mechanisms in human-relevant models exposed to realistic mechanical and matrix conditions. Therapeutic translation will require selectivity, local or tissue-aware delivery, pharmacokinetic testing, and long-term safety assessment, because physiological condensates are essential for normal cell function.
